# A paramedic treatment for modeling explicitly solvated chemical reaction mechanisms†
†Electronic supplementary information (ESI) available. See DOI: 10.1039/c8sc01424h


**DOI:** 10.1039/c8sc01424h

**Published:** 2018-05-30

**Authors:** Yasemin Basdogan, John A. Keith

**Affiliations:** a Department of Chemical and Petroleum Engineering , University of Pittsburgh , Pittsburgh , Pennsylvania 15260 , USA . Email: jakeith@pitt.edu

## Abstract

We report a static quantum chemistry modeling treatment to study how solvent molecules affect chemical reaction mechanisms without dynamics simulations. This modeling scheme uses a global optimization procedure to identify low energy intermediate states with different numbers of explicit solvent molecules and then the growing string method to locate sequential transition states along a reaction pathway. Testing this approach on the acid-catalyzed Morita–Baylis–Hillman (MBH) reaction in methanol, we found a reaction mechanism that is consistent with both recent experiments and computationally intensive dynamics simulations with explicit solvation. In doing so, we explain unphysical pitfalls that obfuscate computational modeling that uses microsolvated reaction intermediates. This new paramedic approach can promisingly capture essential physical chemistry of the complicated and multistep MBH reaction mechanism, and the energy profiles found with this model appear reasonably insensitive to the level of theory used for energy calculations. Thus, it should be a useful and computationally cost-effective approach for modeling solvent mediated reaction mechanisms when dynamics simulations are not possible.

## Abstract 2

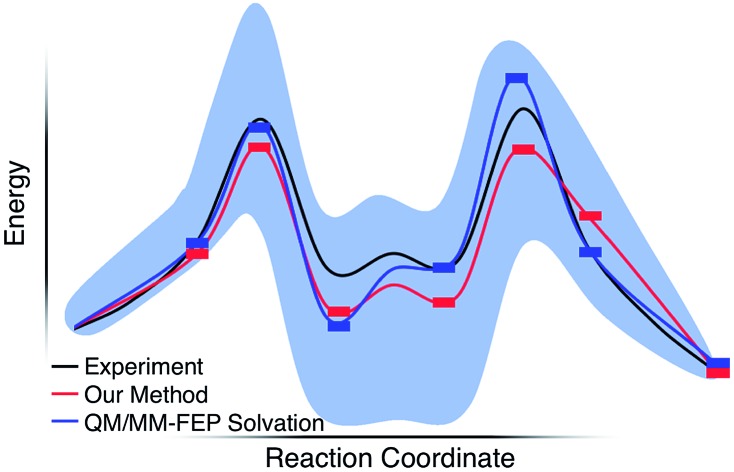
A static QM procedure for modeling solvated reaction mechanisms is calibrated using the Morita–Baylis–Hillman reaction.

## Introduction

Computationally modeling atomic scale chemical reaction mechanisms in solvents is often not trivial. The most reliable and robust schemes usually involve dynamics-based treatments with explicit solvation models, *e.g.* metadynamics,^1^ transition path sampling,^2^ or umbrella sampling^3^ schemes that involve large numbers of electronic structure, QM/MM, or molecular mechanics calculations. While such efforts can be very insightful, they can also bring very large computational costs and/or technical challenges that restrict their use. In contrast, many prefer using computationally inexpensive static quantum chemistry schemes with continuum solvation models (CSMs),^4^*e.g.* the SMD^5^ or COSMO^6^ models. Recent developments of CSMs under periodic boundary conditions^7^–^9^ have excitingly opened avenues for efficient atomic scale studies of reaction mechanisms at solid/liquid interfaces as well.

However, Plata and Singleton's detailed study of the Morita–Baylis–Hillman (MBH) reaction^10^ has underscored poor performances of CSM-based quantum chemistry modeling without explicit solvation. Harvey and Sunoj have since evaluated various quantum chemistry modeling schemes and assembled a mechanistic picture that agrees well with Plata and Singleton's reported mechanism.^11^ Their calculations used the high-level correlated wavefunction method DLPNO-CCSD(T)^12^–^15^ for electronic energies and usually an explicit solvation treatment with molecular mechanics. (They used a CSM treatment in systems where molecular mechanics was not possible). These two important studies have explained the elementary steps of the acid catalyzed MBH reaction mechanism, demonstrated the importance of critically evaluating computational theory to experiment, and discussed the extent that computational modeling can be predictive.

Building from those studies, we now show how one can model such a mechanism with an automatable and paramedic^16^ modeling procedure that is enhanced with chemical intuition but also lessens the need for it. The paramedic and static quantum chemistry procedure will be more computationally demanding than static studies using a CSM with no explicit solvent, but it can also be expected to require less computational effort than many dynamics-based schemes (see below). We formulated the procedure by calibrating to previously reported studies on the MBH reaction in order to understand how to navigate modeling pitfalls that face static models for reaction mechanisms in solvents.

We first assumed that high-level DLPNO-CCSD(T) theory with a relatively large triple-zeta basis set should provide fairly accurate gas phase reaction energies. Thus, any apparent errors larger than a few kcal mol^–1^ in any reaction step would indicate significant errors in solvation energies. We note that interpreting results from CSMs is not trivial, and some have explained that special care is needed.^17^ A standard remedy for inaccurate CSM calculations has the modeler add one or more explicit solvent molecules to the modeled system to more physically describe charge densities and solute–solvent interactions.^18^–^20^ Unfortunately, knowing how many and where solvent molecules should be added is also not trivial unless one makes *a priori* assumptions about local solvent environments. Below, we show that the paramedic model is an automatable way to overcome these challenges.

**Fig. 1:**
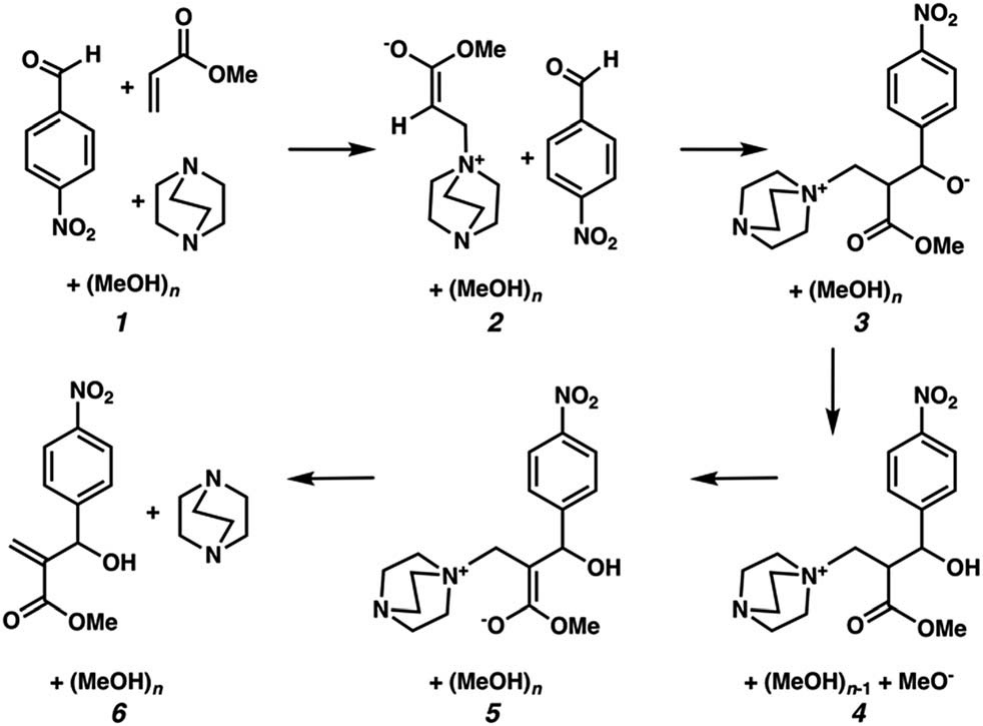
Mechanistic steps for the alcohol mediated MBH reaction, analogous to steps given in ref. 10.

We hypothesized that solvation energies from CSM models would improve if we systematically added explicit methanol molecules around each solute while taking special care to ensure that each microsolvated state was a reasonable approximation of a thermodynamically low energy structure. To test this, we modeled each intermediate from Plata and Singleton's MBH reaction scheme (Fig. 1) as a microsolvated cluster of solutes with *n* = 1, 2, 3, 4, 5, and 10 methanol molecules. In each case we used a stochastic computational filtering procedure using the global optimization code, ABCluster,^21^ to identify low energy geometries (see the ESI† for details). With sufficient searching, a reasonable estimate of the global minimum structure was assumed to be found.

All subsequent quantum chemistry calculations were run using ORCA.^22^ The low energy geometries obtained from the filtering procedure were then fully optimized with BP86-D3/Def2-SVP^23^–^25^ calculations. Hessian calculations at the same level of theory on the lowest energy states confirmed these structures were stationary points and provided free energy contributions from the ideal gas, rigid rotor, and harmonic oscillator (IGRRHO) approximations. Single point electronic energies on these geometries were computed using DLPNO-CCSD(T)^12^–^15^/Def2-TZVP^25^ gas phase energies using solvation energies from the SMD solvation model (using B3LYP-D3/Def2-TZVP calculations and default parameters for methanol solvent). Fig. S1 in the ESI† shows that differences between SMD and COSMO solvation models in these cases are small.

## Results and discussion

One might assume that intermediate **1** is not a good initial reference point for a reaction mechanism since reactant molecules are not infinitely separated and thus are interacting. Indeed, the free energy to form this cluster in gas phase (using free energies from IGRRHO approximations) was calculated to be rather high (about 16 kcal mol^–1^ uphill). To determine the free energy to form the cluster **1** from separated reactants in methanol solvent, we started with the lowest energy structure of **1** and then performed umbrella sampling simulations with classical forcefields with TINKER^26^ to determine a quasi-static pathway that resulted in separated intermediates. Simulations used a cubic box starting with 500 solvent molecules. Dynamics simulations were run where the three intermediates were constrained at incremental intermolecular distances ranging from about 4 to 15 Å (see Fig. S2 and the ESI† for more details). We then used the two-dimensional weighted histogram analysis method^27^ to calculate the free energy profile along this pathway. We found a negligible free energy difference of about 1 kcal mol^–1^ to separate the three solvated reactants across this range of distances (see Fig. S3†). This confirms that this microsolvated cluster is in fact an appropriate reference point for the MBH reaction mechanism study. Future studies will help show if this is generally true for other microsolvated clusters of intermediate states in other reactions.

**Fig. 2:**
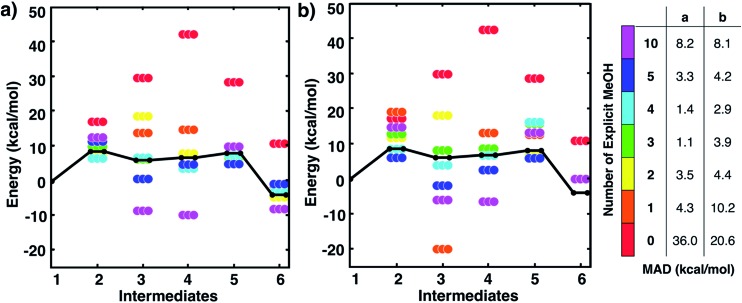
Free energies for MBH reaction intermediates (not including barriers) relative to intermediate **1**. Experimental data (black line) taken from ref. 10. Data with ‘0’ explicit solvent used a calculation scheme with SMD continuum solvation energies, analogous to ref. 10. Relative free energies of clustered intermediates (a) without continuum solvation and (b) with continuum solvation. Mean absolute deviations (MAD, in kcal mol^–1^) compared to experiment are reported in the table on the right. Energies are also tabulated in Tables S1 and S2 of the ESI.†

Fig. 2 shows static quantum chemistry calculation data for each MBH reaction intermediate with different numbers of explicit methanol molecules. States labeled in red use a free energy calculation scheme with SMD continuum solvation and no explicit methanol molecules, analogous to the CSM-based model used in Plata and Singleton's study but now using DLPNO-CCSD(T)/Def2-TZVP electronic energies. Energies in Fig. 2 are Gibbs free energies referenced to **1**. We note that once intermediates are clustered together, the relative free energies are quite similar to their respective relative electronic energies because the zero-point energies and other free energy contributions from IGRRHO approximations with the same number of atoms are similar (see Fig. S4† for comparison of electronic and free energies). Fig. 2 thus showed that our initial hypothesis that gradually adding more solvent molecules into the system would improve agreement to experiment was categorically false. In fact, different numbers of explicit solvent molecules affect different states inconsistently. Furthermore, we unexpectedly found that including solvation energies *via* CSMs generally did not lower mean absolute deviation (MAD) to experimental data in any case compared to their respective gas phase calculations. Interestingly, gas phase microsolvated clusters with just three explicit methanol molecules had the smallest overall MAD, but we assumed this was due to fortuitously error cancellation since adding more methanol molecules usually resulted in less agreement with experiment rather than improved agreement.

**Fig. 3:**
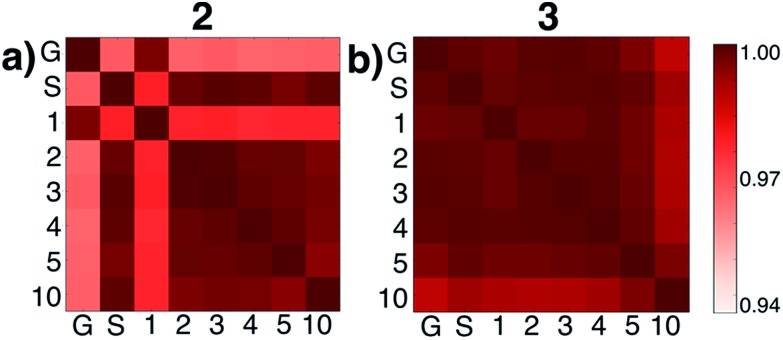
ReMatch-SOAP analysis on the solutes for clusters 2 and 3 with 0 methanol molecules (‘G’ represents a gas phase optimized structure and ‘S’ represents a structure optimized with SMD model), as well as 1, 2, 3, 4, 5, and 10 explicit methanol molecules. Colored boxes quantify similarities in different geometric structures (darker colors represent more similar structures).

We then tested what might be causing errors that we attributed to solvation energy contributions. We hypothesized that different microsolvated clusters might have significantly different solute structures that then reflected different energies shown in Fig. 2. To determine this, we analyzed the geometric similarities of solute structures using the Glosim^28^ algorithm and the ReMatch-SOAP^28^ kernel. Fig. 3 shows a SOAP analysis for intermediates **2** and **3**. The SOAP analysis of different structures for **2** (Fig. 3a) shows very high geometric similarities for all solute structures when gas phase optimizations were run with two or more explicit methanol molecules or when the structure with no explicit solvent was optimized using the SMD model.

In these cases, the C–N bond formed in the initial reaction step had a similar length (*R*_C–N_ = 1.60 Å). The gas phase optimized structures with and without one explicit methanol molecule had a significantly longer and unrealistic C–N bond length (*R*_C–N_ = 2.72 Å), showing those states had fallen downhill in energy into states best described as higher energy conformations of structure **1**. The SOAP analysis of structures for **3** (Fig. 3b) showed the solute geometries within all the microsolvated clusters were highly similar regardless of solvent model. For the intermediate states **4** and **6**, the solute structures having two or more explicit methanol molecules were geometrically very similar to each other. The solute structures for **5**, however, were all found to be dissimilar across all cluster sizes (see Fig. S5†) and could not be interpreted. Apart from this exception, intermediates **2**, **3**, **4**, and **6** all had very similar respective solute structures (regardless of their respective microsolvated cluster sizes ranging from two to 10 methanol molecules). This shows that the 10–30 kcal mol^–1^ scatter in energies for each intermediate shown in Fig. 2 is due to modeling errors in solvation energy contributions that arise in static quantum chemistry calculations that all involve relatively small numbers of explicit solvent. Errors are present whether or not CSM modeling is used, but errors appear to be usually be larger when CSM models are used.

To minimize errors in solvation energies arising from dissimilar local solvation structures, we then modeled the first bond-formation step of the MBH mechanism using Zimmerman's single-ended growing string method (GSM).^29^–^32^ We modeled pathways arising from the lowest energy configurations at each specific degree of solvation that was found using our filtering approach. Of course, one could also straightforwardly use this approach to model multiple pathways starting from different configurations of a single intermediate at the same specific degree of solvation (preferably with the smallest number of explicit solvent molecules). The only limitation to doing this is the higher computational cost of running multiple GSM calculations for each elementary step instead of just one.

All GSM pathway searches were performed with BP86-D3/Def2-SVP calculations with no CSM. We then calculated DLPNO-CCSD(T)/Def2-TZVP gas phase energies for the structures obtained from GSM calculations. Interestingly, transition states for systems with *n* = 1, 3, 4, and 10 methanol molecules each resulted in unreasonably large barriers (∼40 kcal mol^–1^), indicating an unphysical aspect with those microsolvated models for this step (more discussion below). Recall that the case with three methanol molecules also had the lowest MAD in reaction energies in Fig. 2. In cases with *n* = 2 and 5 methanol molecules, more reasonable barriers of 23.6 kcal mol^–1^ and 17.6 kcal mol^–1^ barrier were found.

Closer analysis revealed two points. First, the calculations with two and five methanol molecules both had explicit solvation interactions simultaneously at the two O atoms in **2** that undergo a tautomerization when forming **3**, while the other cases did not simultaneously solvate these two O atoms. (Note that these two atoms were also intuitively solvated by Harvey and Sunoj in their microsolvation models^11^). Second, not only was the barrier with five methanol molecules energetically lower in energy, but it yielded the correct (*S*,*R*) isomer that was discussed in recent mechanism studies. The reaction with two methanol molecules had a higher barrier and resulted in an (*S*,*S*) isomer. Thus, the model with five explicit methanol molecules was the only case out of all solvation models considered that reasonably agreed with known experiment, and this model also resulted in a barrier height in reasonably close agreement with experiment (our calculation: 17.6 kcal mol^–1^, experiment: 20.2 kcal mol^–1^). For these reasons, this model system was the only microsolvated system used further.

We find that explicit solvation interactions are essential for an energetically feasible reaction pathway. However, modeling these interactions does not guarantee an experimentally observable pathway. Using this automatable modeling procedure allows one to see how and the degree that different explicit solvent configurations affect the same reaction step. It is also quite promising that the configuration resulting in the lowest energy pathway also yielded the same stereochemistry as found in previous studies. We see no reason why the configuration involving five methanol molecules is uniquely suited for this step, and one should at this point expect that alternate configurations with different numbers of explicit solvent molecules would also be in play and result in similar energy profiles. We reiterate that sampling solvent configurations with dynamics is the most comprehensive way to understand reaction pathways, but this automatable and all-QM calculation approach can find relevant pathways with less computational effort than that needed for dynamics simulations.

**Fig. 4:**
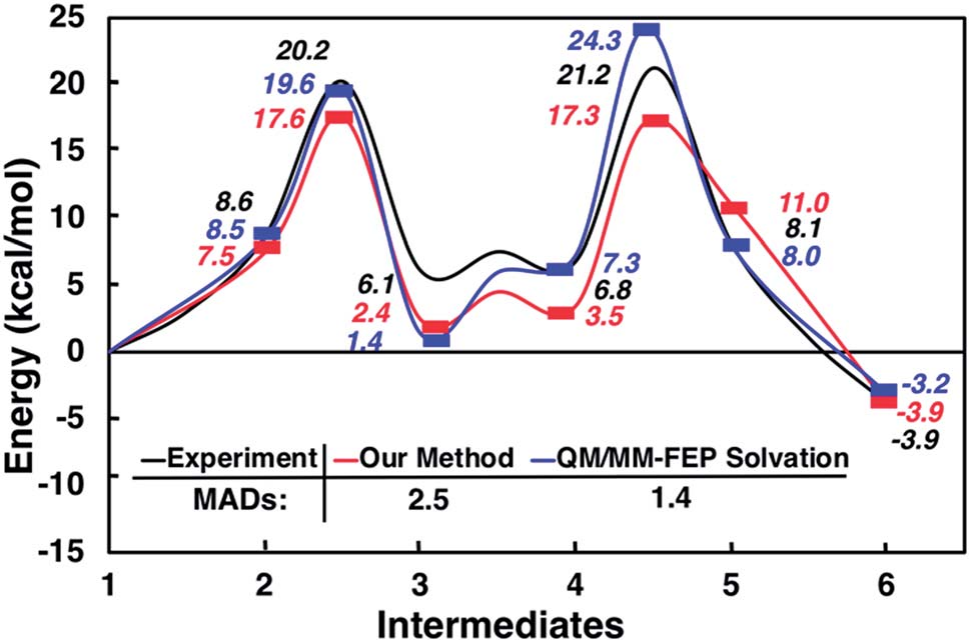
Reaction pathways obtained from GSM calculations (in red) compared to experimental data from ref. 10 (in black) and calculated energies from static and dynamics-based studies from ref. 11 (in blue).

Interestingly, this model for **2** → **3** resulted in a product state, **3′**, that was significantly higher in energy (+7.5 kcal mol^–1^) relative to 3, the state that was found from our global optimization procedures. Not only was the barrier for **2** → **3′** (**TS_2–3′_**) in reasonable agreement with experiment, but the overall energy of **3′** was also in better agreement with experiment (calculated = 2.4 kcal mol^–1^, experiment = 6.1 kcal mol^–1^). Another single-ended GSM calculation starting from **3′** was run to model the proton shuttling reaction that Plata and Singleton rationalized to be very fast,^10^ and this yielded a nearly barrier-less process leading to **4′**, which had reasonable energetics in agreement with experimental data (calculated = 3.5 kcal mol^–1^, experiment = 6.8 kcal mol^–1^). An additional single-ended GSM calculation found the pathway that tautomerized **4′** into its enol form 5′. The calculated barrier (**TS_4′–5′_**, calculated = 17.3 kcal mol^–1^) was also in reasonable agreement with experimental data (experiment = 21.2 kcal mol^–1^), and the tautomerizing O atoms were again simultaneously interacting with explicit methanol molecules in this model. The relative energy of **5′** was in reasonable agreement with experiment (calculated = 11.0 kcal mol^–1^, experiment = 8.1 kcal mol^–1^), while the energy of intermediate **6** was in very close agreement with experiment (calculated = –3.9 kcal mol^–1^, experiment = –3.9 kcal mol^–1^). Fig. 4 summarizes our calculated pathway using five explicit methanol molecules and compares these data to the best calculated data from Harvey and Sunoj's study that used a combination of data from explicit and CSM solvation models. Hence, we have demonstrated a non-conventional, static, and automatable modeling scheme that identifies a complicated reaction mechanism with comparable accuracy as models using computationally demanding explicit solvation methods.

**Fig. 5:**
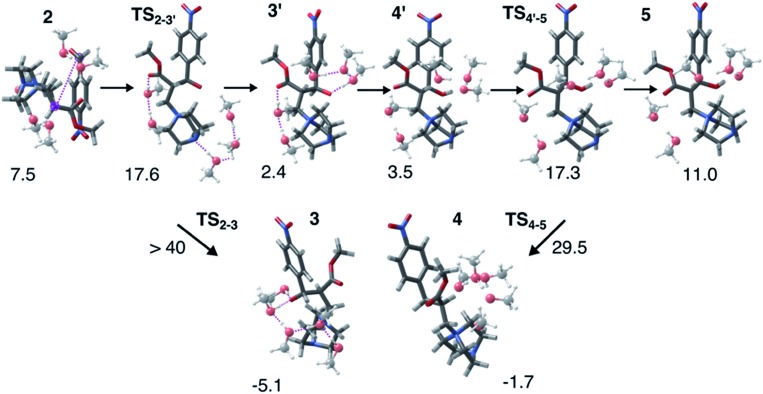
Structures for the MBH reaction pathway (**2** → **TS_2–3′_** → **3′** → **4′** → **TS_4′–5_** → **5**) highlighting the importance of local solvation stabilizing tautomerizing O groups. Though **3′** is calculated to be higher in energy than **3**, explicit hydrogen bonding opens a kinetically feasible pathway for C–C coupling. Reaction energies are reported relative to 1.

As another test for this MBH mechanism, we also ran double ended GSM^29^,^30^ calculations to identify barriers for **3′** → **3** and **4′** → **4** processes. Both barriers were greater than 28 kcal mol^–1^ and would be considered kinetically prohibited within this model. Fig. 5 and S6† summarize these reaction intermediates and calculated data. Lastly, Fig. S7† shows that reaction energies obtained using BP86-D3/Def2-TZVP (MAD = 3.7 kcal mol^–1^) and B3LYP-D3/Def2-TZVP (MAD = 3.1 kcal mol^–1^) single point energies are actually respectably similar to DLPNO-CCSD(T)/Def2-TVP calculations (MAD = 2.5 kcal mol^–1^) as well as far less computationally demanding. We have thus shown calculation schemes using three very different levels of computational theory that are all consistent with each other and are significant improvements over results using a CSM with no explicit solvation. Consistent with our previous study on CO_2_ reduction by borohydride in water,^33^ when modeling sufficiently microsolvated intermediates and transition states, there are only small differences between generalized gradient approximation (GGA) density functional theory, hybrid density functional theory, and high level wavefunction theory calculations. This suggests that even in solution phase reactions as complex as the MBH reaction mechanism, solvation energies are the most critical while electronic energy contributions from high level and computationally intensive methods may be less critical.

## Conclusions

We have demonstrated a new, automatable, and paramedic, modeling scheme that reasonably models the MBH reaction mechanism and should be applicable for studying other challenging reaction mechanisms where CSM models can fail. No dynamics simulations are needed in this model, and transition states are automatically and efficiently found using GSM methods. Four points warrant consideration:

1. As has been stated before by others, CSMs can inadequately model significant local solvation effects in reaction mechanisms, and this affects not only proton shuttling mechanisms but also intramolecular charge transfers or tautomerizations. Remedying this requires some degree of explicit solvation.

2. The degree of explicit solvation required can be probed using this paramedic method that takes advantage of a globally optimized reactant state and error cancellation when modeling reaction pathways as a chronological sequence of GSM pathways.

3. The local solvent environment around a solute plays a critical role in stabilizing reaction intermediates, but any particular solvent environment should neither be assumed to be the same for all intermediates in a reaction mechanism nor easily transferable to different intermediates. We therefore encourage future efforts to report pathways that involve a globally optimized intermediate state followed by a sequence of reaction paths calculated using the growing string method.

4. Once a complete reaction pathway is found using the paramedic method, there appears to be only a marginal gain in accuracy when using high level methods, so a relatively efficient approach such as BP86-D3/Def2-TZVP//BP86-D3/Def2-SVP is likely adequate for qualitatively accurate mechanism predictions and comparison to experiment.

We now summarize steps taken for the paramedic method and recommend that others consider using it to model other reaction mechanism in solvents where CSM models appear to fail. Note that we do not use CSMs in any step of the procedure used here, but in other situations using a CSM might help.

• Step 1: identify globally optimized clustered states for hypothetical reactant states with different numbers of solvent molecules. Our umbrella sampling simulations using explicit solvent models suggest these are adequate representations of reactant states.

• Step 2: systematically explore reaction pathways using single-ended GSM calculations and eliminate models that give unrealistic barriers for processes known to occur and identify a microsolvation model that yields reasonable reaction profiles using energies from a trusted level of computational theory, *e.g.* BP86-D3/Def2-TZVP//BP86-D3/Def2-SVP or DLPNO-CCSD(T)/Def2-TZVP//BP86-D3/Def2-SVP when higher accuracy is needed.

• Step 3 (optional): use double-ended GSM calculations to identify any barrier heights between metastable intermediate states of interest for a complete mechanistic picture.

When successful, this paramedic treatment should be a robust and automatable way to model other challenging reaction mechanisms that involve explicit solvent molecules. Though the paramedic treatment is a multistep process that involves testing variable numbers of explicit solvent molecules, the static quantum chemistry calculations used here are significantly fewer and less computationally demanding than reaction dynamics simulation methods using quantum chemistry. In fact, the slowest step on our study were the single point DLPNO-CCSD(T) calculations. Furthermore, by clustering all atoms into a single microsolvated state, relative free energies (based on IGRRHO approximations) can usually be assumed to closely parallel electronic energies. Thus, hessian calculations might be considered unnecessary as long GSM calculations (which do not involve hessian calculations) correctly identify stationary points. Thus, this paramedic approach appears to capture essential physical chemistry of chemical reactions involving solvent molecules, it appears relatively insensitive to levels of theory used, and it should be considered as a practical alternative to dynamics based computational studies in future studies. Future work will focus on the predictive power of this model on other reactions.

## Conflicts of interest

There are no conflicts of interest to declare.

## Supplementary Material

Supplementary information
