# Supplementary material for: A paramedic treatment for modeling explicitly solvated chemical reaction mechanisms
Source: Chem Sci. 2018 May 30;9(24):5341–6. doi: 10.1039/c8sc01424h (PMC6011202; doi:10.1039/c8sc01424h)
Supplement: Supplementary file 1 [file SC-009-C8SC01424H-s001.pdf]

Electronic Supplementary Material (ESI) for Chemical Science.
This journal is © The Royal Society of Chemistry 2018

Supporting Information

A Paramedic Treatment for Modeling Explicitly Solvated Chemical Reaction Mechanisms.

Yasemin Basdogan and John A. Keith*

Department of Chemical and Petroleum Engineering, University of Pittsburgh, Pittsburgh, Pennsylvania 15260, United States

Table of Contents

1) Methodology S2

2) Comparison of COSMO and SMD Solvation Models S3

3) Umbrella Sampling Simulations S4

4) Comparison of free energies and electronic energies..S6

5) SOAP Analysis on Intermediates *4* and *5* S8

6) Reaction energy profiles involving five explicit methanol molecules S9

7) Reaction energy profiles for intermediates and transition states models with five explicit methanol molecules calculated with different levels of theory. S10

8) Cartesian coordinates of the structures mentioned in main text S11

Structures without clustering (labels follow Ref. 16) S11

Clusters with five methanol molecules (labels follow main text) S14

Clusters with 10 methanol molecules (labels follow main text)....................................S27

1) Methodology

In our study of the Morita Baylis-Hillman (MBH)[1] reaction mechanism, we used a filtering procedure where a global optimization code (ABCluster[2]) automatically generated 1,000 low energy candidates using CHARMM forcefield parameters from MacKerell's CGenFF website.[3] The 100 lowest energy structures from these cases were further optimized using semiempirical PM7[4] optimizations with MOPAC.[5] The five lowest energy structures from these cases were then optimized using Kohn-Sham density functional theory (DFT) at the BP86[6]-D3BJ[7]/Def2-SVP[8] level of theory with ORCA.[9] From now on, '-D3BJ' is shortened to '-D3'. We then compared the lowest energy QM-optimized structure using single point electronic energies using the same BP86-D3 level of theory, a hybrid functional (B3LYP[10]-D3), and a high level ab initio method (DLPNO-CCSD(T)), [11–14] each using the relatively large Def2-TZVP[8] basis set. Calculations made use of RI[15] and RIJCOSX[15] approximations when appropriate. We tested calculations accounting for extended solvation contributions using the SMD model (using B3LYP/Def2-TZVP calculations). We tested both SMD[16] and COSMO[17] solvation models and both provided effectively similar results.

Our calculated reaction energies with no explicit solvent molecules followed an analogous procedure used by Plata and Singleton, where continuum solvation models were used and where thermal and entropic contributions for each solute was obtained using the full standard ideal gas, rigid rotor, and harmonic oscillator approximations.18 Low energy clusters with explicit solvent molecules were made for the six intermediates shown in Scheme 1 of the main text. Vibrational frequency calculations were carried out for the clusters with different number of methanol molecules to confirm there were no imaginary frequencies. Since each clustered intermediate had the same number and type of atoms, the vibrational, thermal, and entropic energy contributions from standard ideal gas, rigid rotor, and harmonic oscillator approximations can be expected to largely cancel out. In general, ΔE and ΔG values agree within about 6 kcal/mol, and these are differences that are much smaller than deviations shown from quantum chemistry modeling that does not account for explicit solvation.

2) Comparison of COSMO and SMD Solvation Models

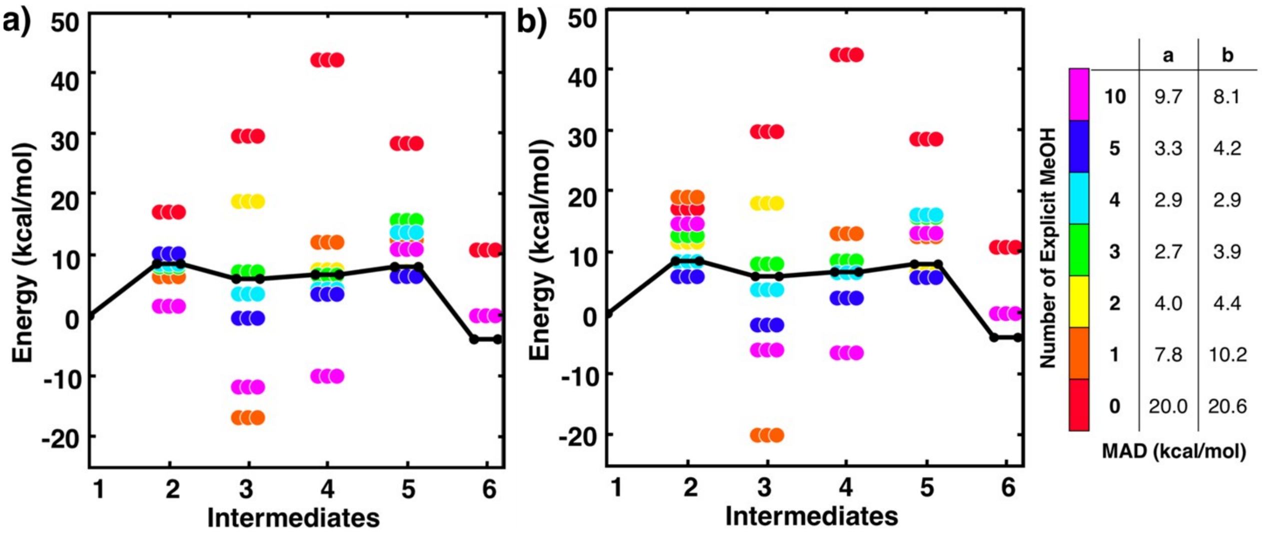

Figure S1: Energies for MBH reaction intermediates (not including barriers) relative to intermediate ***1***. Experimental data (black line) taken from Ref. 1. Relative (a) free energies with COSMO continuum solvation and (b) free energies with SMD continuum solvation. Mean absolute deviations (MAD, in kcal/mol) compared to experiment are reported in the right table.

3) Umbrella Sampling Simulations

All molecular dynamics (MD) simulations for the umbrella sampling were carried out using the TINKER[19] software. The simulations were carried out at 298 K in the NVT ensemble for 2 ns with 1 ps step size where the first 400 ps were used for equilibration and the remaining 1600 ps were used for data collection. In total 60 MD simulations were performed to scan the potential energy surface. In these simulations two constraints were in place: the distances between p-nitrobenzaldehyde and MA and p-nitrobenzaldehyde and DABCO. This was done by defining a harmonic potential between the center of masses of these molecules using a force constant of 100 kcal/mol. For each simulation, the distance between the molecules were varied between ~4 to ~15 Å with a step size of 0.2 Å. At the end of the simulations, the distances were then calculated and the WHAM[20] analysis was carried out.

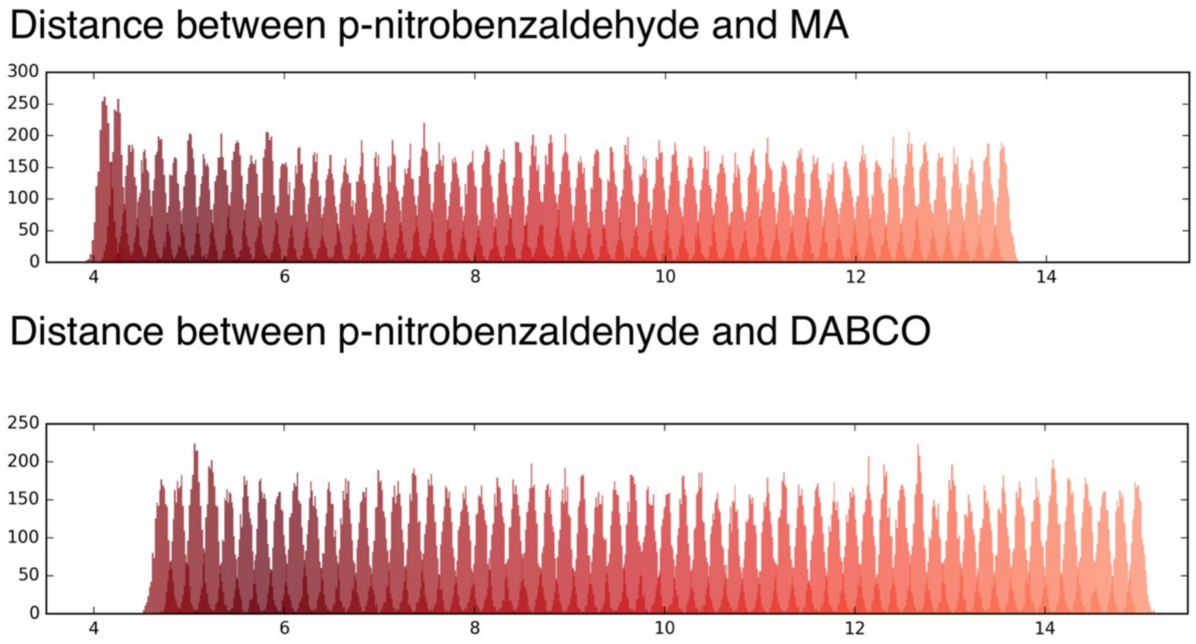

Figure S2: Overlap between umbrella sampling windows for aggregating different molecules together. Distances are defined as the distance between centers of mass for the three different molecules. The overlap of each window shows adequate sampling along the pathway.

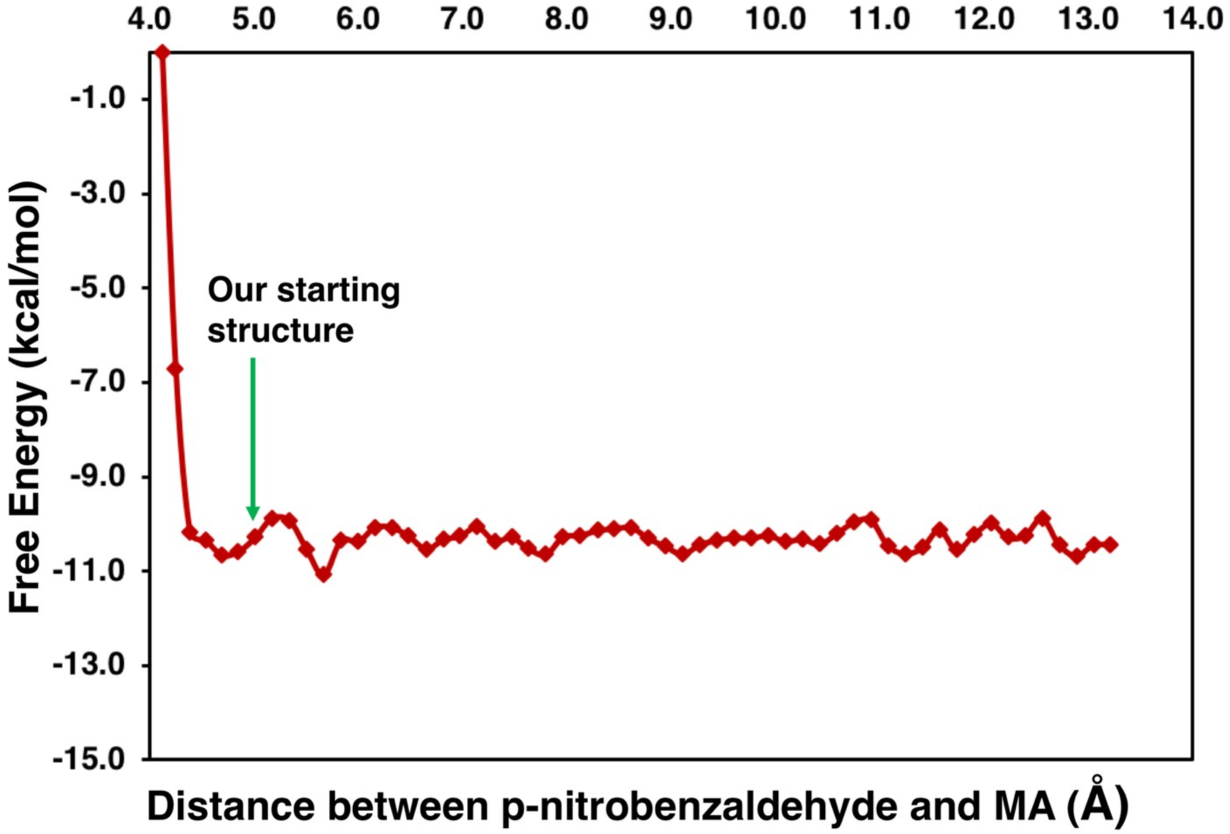

Figure S3: Free energy plot for aggregating three reactant species together. Distances in this chart are defined as the distance between centers of mass for p-nitrobenzaldehyde and MA, but simulations constrained distances between p-nitrobenzaldehyde and MA as well as p-nitorbenzaldehyde and DABCO simultaneously.

4) Comparison of free energies and electronic energies

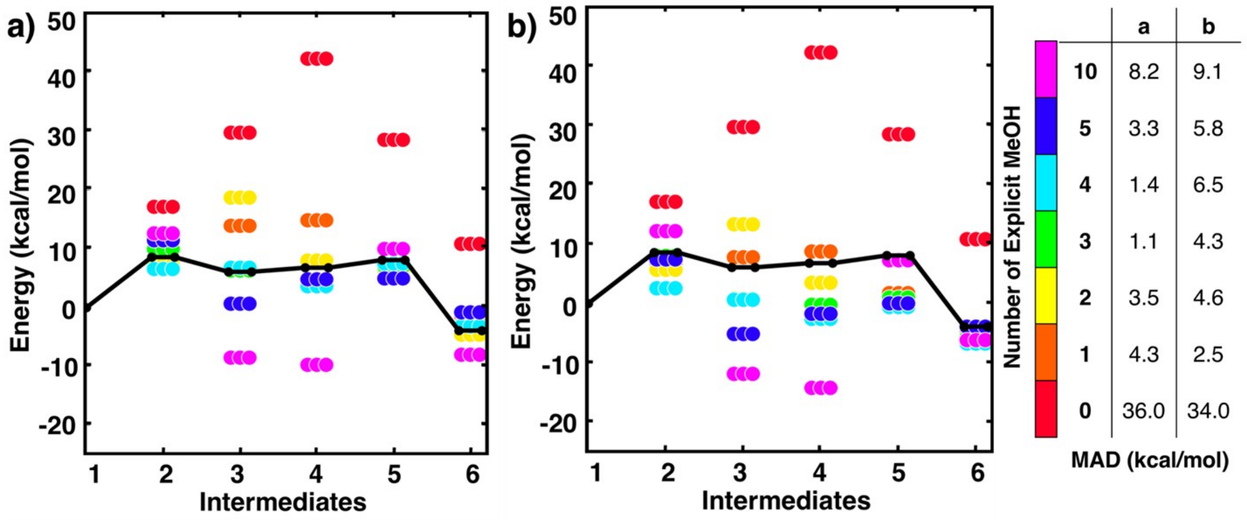

Figure S4: Energies for MBH reaction intermediates (not including barriers) relative to intermediate ***1***. Experimental data (black line) taken from Ref.1. Data with '0' explicit solvent used a calculation scheme with SMD continuum solvation energies, analogous to Ref.1. All remaining calculations are in gas phase. Relative (a) free energies and (b) electronic energies of clustered intermediates. Mean absolute deviations (MAD, in kcal/mol) compared to experiment are reported in the right table.

Table S1. Free energies of clustered intermediates for microsolvated clusters relative to intermediate 1.

# of explicit	Intermediates					
MeOH	1	2	3	4	5	6
0	0	12.1	26.5	152.7	18.0	3.5
1	0	8.12	14.0	14.9	5.2	-1.4
2	0	7.3	18.7	8.0	6.3	-4.6
3	0	10.0	6.3	4.9	7.0	-2.9
4	0	6.6	6.8	3.7	7.5	-3.2
5	0	11.5	0.7	4.8	4.5	-0.8
10	0	12.6	-8.5	-9.7	10.0	-8.0

Table S2. Free energies of clustered intermediates for clusters solvated using the SMD continuum solvation model relative to intermediate 1.

# of explicit	Intermediates					
MeOH	1	2	3	4	5	6[a]
0	0	17.2	29.8	42.4	28.5	10.9
1	0	19.1	-19.9	13.1	12.6	0.0
2	0	11.7	18.1	8.8	6.8	0.0
3	0	12.8	8.1	8.7	15.7	0.0
4	0	8.5	3.9	6.7	16.2	0.0
5	0	6.1	-1.9	2.5	5.9	0.0
10	0	14.7	-5.9	-6.4	13.2	0.0

[a] Relative energies marked at 0.0 were between +0.05 and –0.05 kcal/mol.

5) SOAP Analysis on Intermediates ***4*** and ***5***

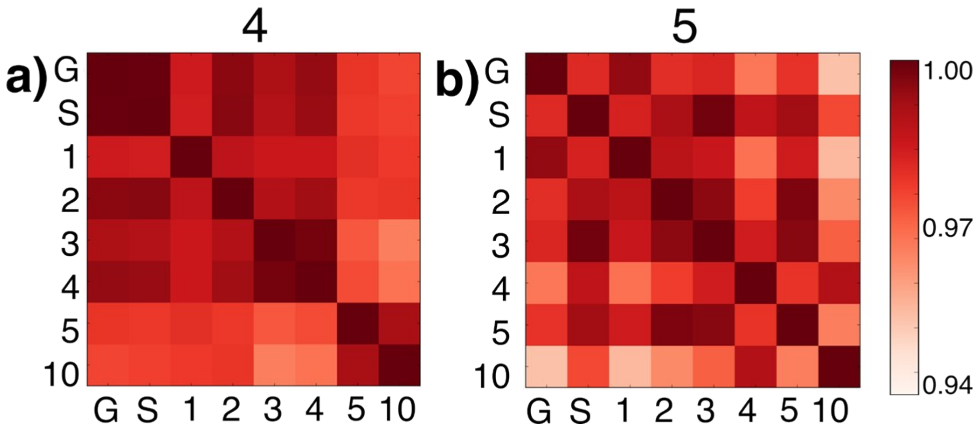

Figure S5: ReMatch-SOAP analysis on the solutes for intermediates ***4*** and ***5*** with no methanol molecules ('G' represents a gas phase optimized structure and 'S' represents a structure optimized with SMD model), as well as one, two, three, four, five, and 10 explicit methanol molecules. Colored boxes quantify similarities in different geometric structures (darker colors represent more similar structures).

6) Reaction energy profiles involving five explicit methanol molecules

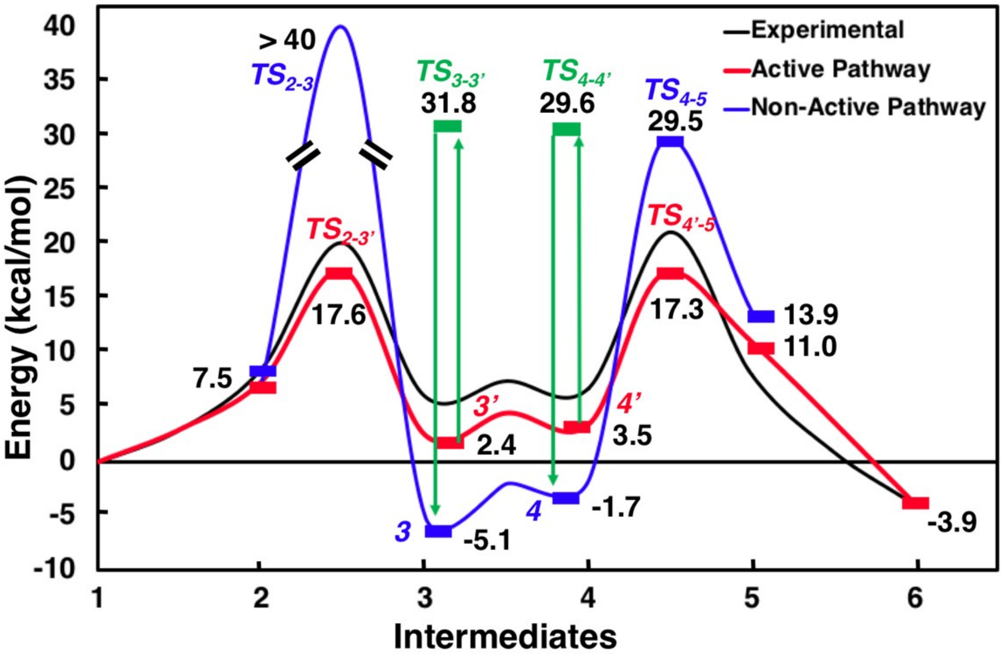

Figure S6: Reaction pathways relative to intermediate ***1*** with different intermediates and transition states obtained from GSM calculations compared to experimental data from Ref. 1. The red line corresponds to the computationally predicted active pathway for the MBH reaction, and the blue line corresponds to a computationally predicted inactive pathway involving low energy (but kinetically inaccessible) intermediates. Energies calculated using the DLPNO-CCSD(T)/Def2-TZVP//BP86-D3/Def2-SVP model chemistry with cluster modeling using five methanol molecules.

7) Reaction energy profiles for intermediates and transition states models with five explicit methanol molecules calculated with different levels of theory.

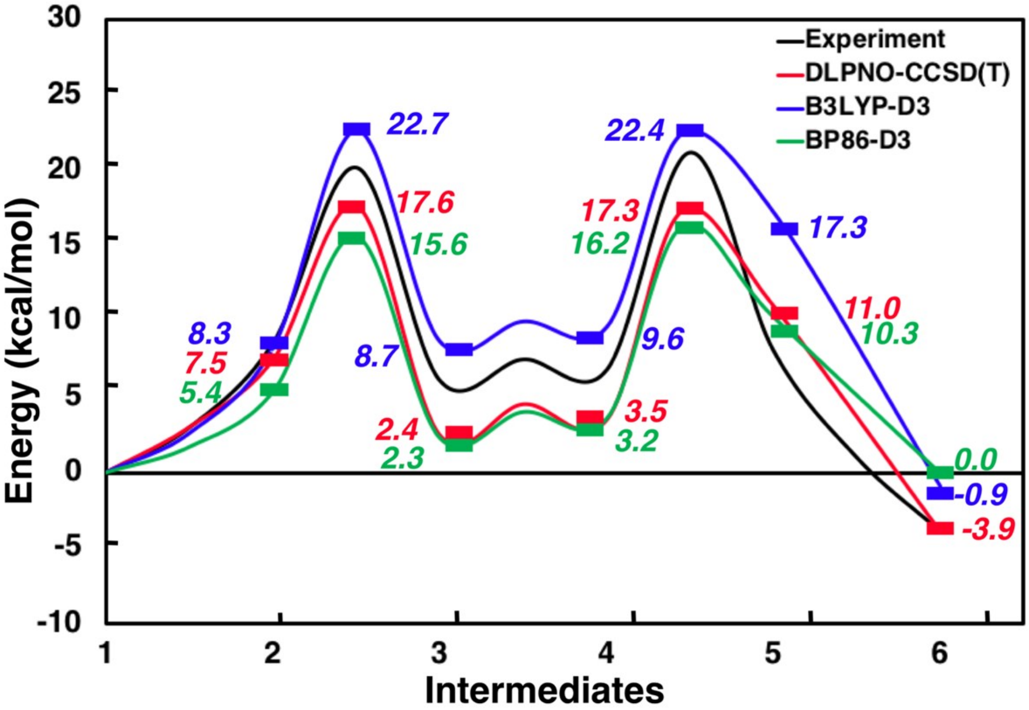

Figure S7: Reaction pathways relative to intermediate ***1*** with different intermediates and transition states obtained from GSM calculations compared to experimental data from Ref.1. Red line corresponds to DLPNO-CCSD(T)/Def2-TZVP//BP86-D3/Def2-SVP, blue line corresponds to B3LYP-D3/Def2-TZVP//BP86-D3/Def2-SVP, and the green line corresponds to BP86/Def2-TZVP//BP86-D3/Def2-SVP model chemistries with cluster modeling using five methanol molecules.

8) Cartesian coordinates of the structures mentioned in main text

Structures without clustering (labels follow Ref. 16)

MA

C	-0.04537	0.00042	0.19350
O	-0.03873	0.00052	1.42062
O	1.08575	0.00017	-0.55181
C	-1.25235	-0.00040	-0.67083
C	2.32424	-0.00007	0.18274
H	3.12764	0.00165	-0.57440
H	2.40196	0.90175	0.82267
H	2.40314	-0.90387	0.81974
C	-2.48749	0.00027	-0.13470
H	-1.08557	-0.00154	-1.75996
H	-3.38918	-0.00036	-0.76601
H	-2.62384	0.00147	0.95982

MeO

C	-0.04995	0.08679	-0.03543
H	0.02442	-0.04195	1.11396
H	1.05925	-0.05365	-0.33870
H	-0.48341	-0.94391	-0.33909
O	-0.67934	1.17621	-0.49217

MeOH

O	0.02569	-0.00006	-0.03522
C	-0.01349	-0.00003	1.38541
H	1.03246	0.00003	1.75479
H	-0.51882	0.90110	1.80794
H	-0.51878	-0.90113	1.80802
H	-0.89989	0.00009	-0.34760

DABCO

N	1.61500	0.25138	-0.64124
C	1.02874	-0.28007	-1.88921
C	2.19913	1.57984	-0.91904
C	2.69302	-0.65768	-0.19881
C	3.79015	-0.77226	-1.30545
C	2.12660	-0.39343	-2.99517
C	3.29828	1.46559	-2.02365
N	3.42946	0.06292	-2.46921
H	3.11389	-0.26183	0.74832
H	2.24402	-1.64597	0.03049
H	2.61761	1.98026	0.02743
H	1.38096	2.26231	-1.23010
H	0.20652	0.39584	-2.20379
H	0.57459	-1.26807	-1.66741
H	3.05293	2.09116	-2.90720
H	4.28546	1.80416	-1.64724
H	2.24000	-1.44008	-3.34545
H	1.87279	0.22264	-3.88262
H	4.77900	-0.43675	-0.93029
H	3.90889	-1.82003	-1.65062

Structure 11

C	4.58502	-0.42999	-0.20487
C	3.87520	-1.62540	-0.42433
C	2.66422	-1.81503	0.24557
C	2.14946	-0.82452	1.10760
C	2.89374	0.35560	1.32458
C	4.11041	0.56504	0.66952
C	0.80177	-1.08246	1.78777
O	0.46269	-2.34823	1.92222
N	5.85405	-0.21767	-0.89751
O	6.45820	0.84818	-0.70814
O	6.27218	-1.10914	-1.65053
C	-0.32230	-0.25901	0.82936
C	-1.65024	-0.62040	1.44617
N	-2.86082	-0.52417	0.53659
C	-2.80174	-1.56765	-0.56148
C	-4.04026	-1.39380	-1.48124
C	-0.04233	1.20338	0.80749
O	0.55522	1.59752	-0.34660

C	0.98766	2.96891	-0.40087
O	-0.25430	1.97586	1.74253
C	-4.10340	-0.77758	1.37130
C	-5.34174	-0.80228	0.43740
C	-2.98280	0.84503	-0.10183
C	-4.32106	0.90991	-0.88887
H	-1.86299	0.02588	2.31980
H	1.42144	3.11168	-1.40628
H	0.13333	3.65906	-0.25373
H	1.75370	3.17370	0.37358
H	-1.56921	-1.67903	1.77306
H	-0.18007	-0.70026	-0.17460
H	0.79243	-0.45053	2.73419
H	2.51702	1.11879	2.02460
H	4.70080	1.47822	0.82276
H	4.28282	-2.38120	-1.10936
H	2.07661	-2.73956	0.13341
H	-1.85115	-1.42236	-1.10203
H	-2.76831	-2.55044	-0.05706
H	-2.92236	1.59010	0.71192
H	-2.10418	0.96756	-0.75905
H	-3.93981	-1.73248	1.90257
H	-4.14404	0.03471	2.12030
N	-4.97771	-0.40470	-0.93031
H	-3.72916	-1.06233	-2.48983
H	-4.56055	-2.36374	-1.59566
H	-5.78330	-1.81653	0.40454
H	-6.11723	-0.11351	0.82361
H	-5.01029	1.63493	-0.41536
H	-4.13021	1.25610	-1.92212

Structure 12

C	4.59886	-0.10198	-0.03688
C	3.91273	-1.19279	-0.59391
C	2.68764	-1.56821	-0.03455
C	2.16049	-0.86387	1.06674
C	2.88091	0.21436	1.62019
C	4.10466	0.60794	1.06786
C	0.77845	-1.20835	1.59687
O	0.47002	-2.58609	1.49602
N	5.88200	0.30808	-0.62790
O	6.47099	1.27390	-0.13146
O	6.30790	-0.33267	-1.59500
C	-0.32254	-0.47610	0.76347
C	-1.66257	-0.66659	1.47015
N	-2.88290	-0.52797	0.58989
C	-2.98600	-1.68625	-0.38595
C	-4.21256	-1.44949	-1.30718
C	0.05377	0.99239	0.61994
O	0.63864	1.23534	-0.56185
C	1.15603	2.57059	-0.76018
O	-0.12062	1.83336	1.49108
C	-4.11466	-0.54882	1.48049
C	-5.38012	-0.56024	0.58386
C	-2.88250	0.77362	-0.19044
C	-4.24665	0.91263	-0.92214
H	-1.76816	0.07590	2.28397
H	1.61946	2.56674	-1.76110
H	0.33604	3.31358	-0.71842
H	1.91050	2.80894	0.01423
H	-1.70850	-1.68274	1.90040
H	-0.30572	-0.95170	-0.23586
H	0.70201	-0.85063	2.64939
H	2.47791	0.75644	2.48846
H	4.67950	1.44698	1.48057
H	4.34017	-1.72303	-1.45430
H	2.12976	-2.42102	-0.44778
H	-2.03956	-1.73723	-0.95115
H	-3.07642	-2.60088	0.22822
H	-2.69736	1.58796	0.53200
H	-2.03913	0.72162	-0.90194
H	-4.03150	-1.44498	2.12127
H	-4.04703	0.34851	2.12265
N	-5.02828	-0.32697	-0.82367
H	-3.88348	-1.22734	-2.33974
H	-4.83175	-2.36607	-1.34526
H	-5.90220	-1.53294	0.66135
H	-6.08362	0.22436	0.92139
H	-4.83518	1.73984	-0.48197

H	-4.07203	1.15590	-1.98703
H	0.99743	-3.06351	2.16728

Structure 20

C	0.76162	0.79225	-0.81856
C	-0.22132	-0.14585	-0.40935
O	-1.51996	0.33696	-0.63871
O	-0.06736	-1.26524	0.12561
C	-2.58222	-0.59522	-0.45177
H	-3.51877	-0.04826	-0.67045
H	-2.49407	-1.46006	-1.14329
H	-2.61229	-0.98147	0.58840
C	2.14370	0.56158	-0.41752
H	2.36601	-0.51310	-0.26693
H	2.90576	1.01445	-1.08221
N	2.55784	1.19068	1.01939
C	2.49713	2.69305	0.95856
H	3.18750	3.01331	0.15598
H	1.46378	2.95690	0.66362
C	2.89583	3.26817	2.34667
C	3.95226	0.76160	1.37401
H	4.61379	1.08144	0.54684
C	4.34275	1.40104	2.73825
H	3.94983	-0.34421	1.40772
C	1.60941	0.72034	2.08720
C	2.05626	1.31939	3.45034
H	0.60506	1.05940	1.77622
H	1.61706	-0.38431	2.06972
C	0.33114	2.05424	-1.52180
H	1.24392	2.63021	-1.79372
C	-1.71423	1.19784	-5.24083
C	-2.28954	2.11681	-4.34835
C	-1.62109	2.40233	-3.15352
C	-0.40079	1.77130	-2.83634
C	0.15872	0.86130	-3.76134
C	-0.48667	0.56881	-4.96466
N	-2.40708	0.88865	-6.49046
H	-3.24769	2.59012	-4.59916
H	-2.04866	3.11893	-2.43827
H	1.10929	0.36437	-3.51712
H	-0.06254	-0.13881	-5.68959
O	-0.44186	2.95136	-0.68738
H	-1.13318	2.36396	-0.30604
O	-1.89717	0.05915	-7.25658
O	-3.47469	1.46943	-6.73252
N	3.23170	2.19119	3.29103
H	4.61716	0.61257	3.46541
H	5.22185	2.06285	2.61597
H	3.76979	3.94074	2.24641
H	2.06360	3.86365	2.77006
H	1.23388	1.91096	3.89739
H	2.30880	0.50918	4.16121

p-nitrobenzaldehyde

C	0.23226	-1.70938	-0.00014
C	-1.04901	-1.11397	-0.03181
C	-1.17377	0.27628	-0.03279
C	-0.00178	1.05152	-0.00012
C	1.28204	0.48856	0.03243
C	1.39203	-0.90618	0.03138
H	-1.93901	-1.75980	-0.05570
H	-2.15388	0.76968	-0.05807
N	-0.12768	2.52330	-0.00018
H	2.16576	1.13838	0.05754
H	2.38538	-1.37990	0.05553
C	0.36934	-3.18685	0.00033
O	-0.57686	-3.96674	-0.02473
H	1.42942	-3.56347	0.02651
O	0.90678	3.19396	0.05553
O	-1.26124	3.00745	-0.05590

Structure 7

C	-2.15939	-0.74005	-0.93895
C	-2.60845	0.17703	0.02345
C	-1.83489	0.51377	1.14603
C	-0.57934	-0.08189	1.29628
C	-0.09971	-1.00582	0.34219
C	-0.90176	-1.32983	-0.76995

C	1.27708	-1.65511	0.51919
O	1.66091	-2.44917	-0.59374
C	2.32387	-0.58810	0.82964
C	2.61704	0.32028	-0.31684
O	2.25655	0.07071	-1.47194
C	2.93027	-0.46349	2.02816
O	3.30272	1.42183	0.01253
C	3.64820	2.30925	-1.07287
H	2.70314	-1.15933	2.85196
H	3.67118	0.32789	2.21849
H	4.21240	3.13954	-0.61440
H	4.27391	1.78364	-1.82102
H	2.73337	2.69446	-1.56397
H	1.21606	-2.34006	1.39181
H	1.85870	-1.79249	-1.30688
N	-3.92457	0.80391	-0.14887
H	-2.21881	1.23374	1.88054
H	0.03847	0.17990	2.16851
H	-0.53006	-2.05421	-1.50821
H	-2.79419	-0.97882	-1.80239
O	-4.59559	0.49170	-1.13928
O	-4.30204	1.61598	0.70408

Structure 8

C	-4.61298	0.86874	0.17674
O	-3.62385	-0.03065	0.67138
C	-2.52060	-0.23196	-0.17339
O	-2.48400	0.36042	-1.27882
C	-1.57133	-1.12955	0.36529
C	-0.38760	-1.45918	-0.40803
N	0.91567	-0.50662	-0.14152
C	1.40444	-0.70695	1.26278
C	0.54714	0.93821	-0.32718
C	2.01454	-0.85713	-1.09985
H	-1.74384	-1.56956	1.35818
H	-5.40004	0.92781	0.95388
H	-4.20043	1.88521	-0.00345
H	-5.06543	0.51253	-0.77413
H	0.04296	-2.46029	-0.20849
H	-0.53280	-1.31463	-1.49757
H	0.10681	1.03715	-1.33577
H	-0.25083	1.14749	0.40859
C	1.81754	1.80779	-0.11721
H	1.62518	-0.66964	-2.11850
C	3.26303	0.01149	-0.76818
H	2.20890	-1.94167	-0.99952
C	2.59933	0.25250	1.52157
H	0.53969	-0.51453	1.92556
H	1.68538	-1.77297	1.35851
H	2.34348	0.99001	2.30635
H	3.47851	-0.31918	1.87723
H	4.10358	-0.62892	-0.43728
H	3.59715	0.55806	-1.67077
N	2.96530	0.98307	0.29688
H	2.08123	2.33916	-1.05254
H	1.63273	2.57611	0.65850

Clusters with five methanol molecules
(labels follow main text)

Intermediate 1

C	13.03388	14.75704	9.95948
C	13.47314	14.87523	8.62141
C	12.78788	14.21952	7.59708
C	11.66073	13.44428	7.93204
C	11.20395	13.31499	9.25523
C	11.89990	13.97522	10.26580
H	14.36411	15.48367	8.40777
H	13.10683	14.29033	6.54883
N	10.93668	12.74671	6.87117
H	10.33059	12.69167	9.47085
H	11.56283	13.91543	11.30974
C	13.75603	15.45141	11.05705
O	14.82437	16.06169	10.89496
H	13.37442	15.19627	12.07942
O	9.89683	12.14004	7.17455
O	11.39014	12.78577	5.71964
N	12.08401	17.12974	11.04794
C	10.82945	16.94855	10.30160

C	12.83094	18.26958	10.50943
C	11.78178	17.33451	12.47330
H	13.81969	18.31033	11.00865
H	13.00897	18.08458	9.43074
C	11.98878	19.57241	10.73822
H	10.28591	16.09922	10.75892
C	9.99787	18.27618	10.40392
H	11.07216	16.69793	9.24967
H	12.73867	17.46665	13.01643
C	10.86012	18.59574	12.61414
H	11.28413	16.41993	12.84536
H	12.50215	20.26017	11.44070
H	11.84412	20.11883	9.78456
H	9.87744	18.74854	9.40843
H	8.98175	18.07266	10.79832
N	10.66840	19.23489	11.30001
H	9.86497	18.31377	13.01340
H	11.30157	19.33543	13.31272
C	11.56109	13.37360	14.23556
O	12.46882	13.26286	13.40309
O	10.51563	12.53721	14.31397
C	11.50659	14.42265	15.27574
C	10.39901	11.54408	13.27984
H	9.40206	11.08857	13.40217
H	10.47845	12.02176	12.28718
H	11.18177	10.76802	13.39183
C	12.49045	15.33404	15.40007
H	10.62390	14.41950	15.93355
H	12.44641	16.12258	16.16717
H	13.37304	15.30573	14.74110
O	9.67809	14.73076	12.45965
H	9.19763	14.13267	11.80941
C	8.70339	15.19843	13.37028
H	9.19095	15.86790	14.10995
H	8.21541	14.37387	13.94187
H	7.89553	15.78800	12.87327
O	11.55485	10.26715	10.11043
H	12.22305	10.70551	10.73565
C	12.17724	10.04384	8.85186
H	11.41501	9.67343	8.13783
H	12.97850	9.27337	8.92401
H	12.62973	10.96727	8.42553
O	13.30664	11.36628	11.71536
H	12.95786	12.08850	12.31229
C	14.54081	11.78818	11.13896
H	15.05975	10.89691	10.73204
H	15.20372	12.25096	11.90160
H	14.40018	12.51463	10.30798
O	8.23075	13.19399	10.87370
H	8.52675	12.24095	10.68451
C	7.70785	13.75743	9.67621
H	7.56526	14.84684	9.82780
H	6.71649	13.32223	9.41459
H	8.38418	13.61898	8.80152
O	9.01375	10.76555	10.35713
H	10.00953	10.59977	10.24126
C	8.31206	10.08435	9.31890
H	7.22339	10.16523	9.51717
H	8.57096	9.00191	9.30162
H	8.51656	10.51321	8.31371

Intermediate 2

C	9.86983	10.50581	6.26604
C	11.00581	9.83522	5.75949
C	11.67937	10.34154	4.64639
C	11.21623	11.53543	4.06839
C	10.09106	12.22059	4.54824
C	9.41216	11.68669	5.64754
H	11.33718	8.90451	6.24137
H	12.55228	9.83570	4.21733
N	11.93122	12.06904	2.89777
H	9.77848	13.17791	4.11377
H	8.55181	12.22619	6.06566
C	9.17683	9.98868	7.47524
O	9.67984	9.17329	8.24385
H	8.12931	10.35985	7.62124
O	11.44450	13.03700	2.30555
O	12.97560	11.50843	2.54711
C	9.68262	16.00518	2.92614

O	10.38738	16.58017	4.02748
C	10.09088	16.07786	5.28952
O	9.16605	15.20329	5.37281
C	10.86925	16.64151	6.30887
C	10.78617	16.19873	7.70181
N	11.72558	14.95545	8.05542
C	13.17030	15.30309	7.83773
C	11.38480	13.80190	7.15110
C	11.52626	14.53246	9.48469
H	9.90440	16.64528	2.05052
H	10.03598	14.97454	2.71273
H	8.58744	15.98126	3.10113
H	10.29556	13.66752	7.17967
H	11.70083	14.08766	6.13431
C	12.13354	12.55492	7.63903
H	10.49366	14.14285	9.54898
C	12.56274	13.42971	9.83003
H	11.63022	15.42982	10.12208
C	14.01687	14.01808	8.00180
H	13.24321	15.72406	6.81802
H	13.43124	16.08863	8.57120
H	14.37876	13.67131	7.01668
H	14.89349	14.20718	8.65022
H	13.35374	13.81213	10.50338
H	12.05984	12.58694	10.34174
N	13.20273	12.93196	8.59450
H	11.43418	11.87813	8.16368
H	12.58470	12.02014	6.78377
H	9.78597	15.82470	7.99736
H	11.11651	16.96276	8.43208
H	11.63376	17.37571	6.01492
O	14.59833	10.74193	8.05427
H	14.12725	11.55182	8.47467
C	13.80746	9.58206	8.29794
H	14.10191	8.78883	7.58056
H	12.71576	9.76672	8.18186
H	13.97357	9.18589	9.32617
O	14.79985	11.55318	5.54796
H	14.71085	11.21941	6.50111
C	15.39403	10.53182	4.76163
H	15.57725	10.92650	3.74256
H	14.74806	9.62844	4.66267
H	16.37237	10.20365	5.18102
O	13.24020	13.56474	4.77843
H	13.88515	12.81470	4.93605
C	13.74857	14.49823	3.85094
H	13.02251	15.33462	3.77393
H	13.87526	14.06329	2.82923
H	14.73320	14.92405	4.15947
O	9.17391	12.33490	8.86629
H	8.65975	13.06104	8.39778
C	8.51644	11.96907	10.06497
H	8.98015	11.03542	10.44548
H	7.42899	11.76872	9.92059
H	8.61236	12.74395	10.86356
O	7.99953	14.28874	7.46632
H	8.51695	14.66938	6.65699
C	6.64037	14.12893	7.07732
H	6.03167	13.89149	7.97419
H	6.49768	13.30111	6.34125
H	6.23123	15.06026	6.62577

$TS_{2-3'}$

C	10.12914	13.54270	3.49438
C	10.95199	13.97762	2.43455
C	10.43829	14.13703	1.14582
C	9.08257	13.83429	0.91992
C	8.24414	13.37135	1.94786
C	8.77893	13.22588	3.23262
H	12.01198	14.17407	2.65352
H	11.06165	14.48948	0.31332
N	8.53120	13.99649	-0.42599
H	7.19576	13.13155	1.72837
H	8.13459	12.86447	4.04815
C	10.73913	13.42509	4.89545
O	12.01699	13.02248	4.88246
H	10.02849	12.76125	5.48056
O	7.33149	13.74361	-0.60447
O	9.28643	14.38380	-1.32792

C	7.93292	16.71695	3.81458
O	9.21816	16.28238	4.30633
C	9.20978	15.42726	5.33832
O	8.15880	15.09005	5.90195
C	10.56893	14.86995	5.64238
C	10.71021	14.84746	7.16920
N	11.96080	14.25181	7.78069
C	13.22024	14.63800	7.03053
C	11.87212	12.73184	7.80498
C	12.05244	14.76041	9.21066
H	7.39990	17.30710	4.58578
H	8.14591	17.34474	2.93218
H	7.31336	15.84558	3.52512
H	10.89415	12.49167	8.25395
H	11.93067	12.44181	6.73183
C	13.05429	12.18007	8.62902
H	11.13888	14.39718	9.71931
C	13.35781	14.23126	9.85168
H	12.03093	15.86414	9.16008
C	14.42257	13.92453	7.70121
H	13.05506	14.26632	5.99558
H	13.29030	15.74094	7.06363
H	14.81383	13.13391	7.02996
H	15.23183	14.64373	7.92729
H	14.06896	15.05554	10.04697
H	13.13916	13.74197	10.82049
N	14.00968	13.25862	8.95752
H	12.70528	11.72014	9.57374
H	13.60446	11.42502	8.04164
H	9.87223	14.28901	7.62251
H	10.66209	15.88719	7.54365
H	11.32280	15.53207	5.17605
O	15.95768	11.74415	9.94909
H	15.25775	12.41892	9.60596
C	15.60943	11.39394	11.28520
H	16.31942	10.62332	11.64905
H	14.58066	10.97356	11.37204
H	15.68239	12.26890	11.97024
O	15.79358	9.74827	8.24941
H	15.91624	10.50598	8.91736
C	14.88398	8.80860	8.81131
H	14.71806	7.99284	8.07845
H	13.89061	9.25191	9.05388
H	15.28700	8.34820	9.74217
O	14.86358	11.05481	6.10454
H	15.24356	10.50006	6.84939
C	13.73733	10.37862	5.56308
H	13.12774	11.11192	4.99194
H	13.06940	9.95211	6.35049
H	14.02853	9.53500	4.88956
O	9.34943	13.15016	9.89621
H	8.67945	13.23025	9.15283
C	9.33617	11.81306	10.37135
H	9.70391	11.07283	9.62009
H	8.32204	11.48520	10.69804
H	10.00546	11.74559	11.25423
O	7.67945	13.25196	7.81175
H	7.85196	13.96975	7.13536
C	7.68980	11.99492	7.13798
H	6.97647	11.98307	6.28475
H	7.36902	11.21153	7.85241
H	8.69957	11.71830	6.75550

Intermediate 3

C	8.80158	14.17854	13.69480
C	8.49990	15.55436	13.59766
C	7.25610	16.04001	14.00985
C	6.30872	15.13229	14.51543
C	6.57469	13.75723	14.61149
C	7.82573	13.28982	14.19393
H	9.25577	16.24509	13.19263
H	7.00650	17.10749	13.94938
N	5.00202	15.63476	14.95130
H	5.80632	13.07694	15.00100
H	8.05217	12.21515	14.26055
C	10.17482	13.66643	13.26667
O	10.63161	14.31527	12.13128
H	10.07901	12.56598	13.09507
O	4.19023	14.82749	15.42098

O	4.77068	16.84465	14.83270
C	9.16623	14.17407	17.53952
O	10.00766	14.45860	16.40326
C	10.49168	13.39058	15.74203
O	10.32320	12.23290	16.10645
C	11.20397	13.79507	14.46318
C	12.43718	12.88089	14.36232
N	13.52667	13.23308	13.37225
C	13.87605	14.70599	13.39644
C	13.15538	12.81555	11.95735
C	14.76564	12.43787	13.76142
H	8.83057	15.15375	17.92021
H	8.29422	13.56376	17.23164
H	9.73351	13.63270	18.32182
H	13.06680	11.71652	12.00580
H	12.16633	13.27601	11.74714
C	14.27071	13.30011	11.00011
H	14.44643	11.37985	13.83377
C	15.84794	12.64511	12.67106
H	15.07946	12.80905	14.75401
C	15.14203	14.92500	12.52389
H	13.00810	15.27140	13.00783
H	14.03717	14.98449	14.45475
H	14.97176	15.77492	11.83489
H	16.01447	15.18159	13.15509
H	16.81695	12.89049	13.14621
H	15.99177	11.71506	12.08797
N	15.46971	13.72228	11.74286
H	14.54289	12.49053	10.29459
H	13.92049	14.15804	10.39220
H	12.11268	11.85390	14.10950
H	12.94336	12.85789	15.34592
H	11.47889	14.86278	14.55910
O	13.48634	9.53956	13.05435
H	12.50897	9.77847	12.99484
C	13.90116	9.06953	11.78350
H	14.95581	8.73128	11.85481
H	13.86400	9.85295	10.98732
H	13.29715	8.20176	11.42865
O	11.48252	16.70682	12.27153
H	11.09933	15.74832	12.24891
C	11.71940	17.06402	10.92460
H	12.23009	18.05106	10.88580
H	10.77801	17.15452	10.32869
H	12.37307	16.33399	10.38527
O	10.98685	10.35644	12.86318
H	10.71272	10.55404	11.89440
C	9.90504	9.73001	13.54123
H	9.90247	8.62417	13.39685
H	8.92462	10.11755	13.18579
H	9.98599	9.94395	14.62706
O	9.43668	13.30655	10.19968
H	9.93522	13.80507	11.00443
C	8.03594	13.49047	10.33581
H	7.52292	13.11926	9.42230
H	7.77459	14.56619	10.45564
H	7.60320	12.94399	11.20738
O	10.24336	10.90021	10.45909
H	9.87636	11.86783	10.37396
C	11.21594	10.72446	9.43882
H	11.62264	9.69462	9.49670
H	12.06837	11.43930	9.51770
H	10.76969	10.85293	8.42614

Intermediate 3’

C	-0.01030	-1.45260	-2.06770
C	-1.15600	-2.25040	-1.84960
C	-1.15980	-3.60190	-2.19550
C	-0.00430	-4.15550	-2.77720
C	1.14400	-3.38710	-3.02030
C	1.12950	-2.03350	-2.65950
H	-2.04100	-1.79950	-1.38240
H	-2.03380	-4.24300	-2.01940
N	-0.00140	-5.57600	-3.13870
H	2.02640	-3.85150	-3.47890
H	2.02570	-1.41760	-2.83030
C	0.00000	0.00000	-1.60170
O	-1.02200	0.74310	-2.12540
H	1.00810	0.41830	-1.87610

O	1.01580	-6.04730	-3.66220
O	-1.01730	-6.24280	-2.90140
C	1.42380	-3.27820	0.96110
O	0.50960	-2.18150	0.76030
C	1.03820	-0.99530	0.44700
O	2.26090	-0.79990	0.44750
C	0.00000	0.00000	0.00000
C	0.33850	1.34390	0.65530
N	-0.64700	2.48150	0.49010
C	-2.08440	2.00170	0.46600
C	-0.37640	3.25850	-0.79080
C	-0.45270	3.43380	1.65940
H	0.79280	-4.17790	1.06320
H	2.10370	-3.37960	0.09300
H	2.02220	-3.12530	1.88020
H	0.64830	3.65460	-0.67810
H	-0.43990	2.50950	-1.60860
C	-1.45620	4.36140	-0.92980
H	0.62800	3.66890	1.68700
C	-1.34040	4.68440	1.43390
H	-0.73190	2.87280	2.57010
C	-3.01150	3.24450	0.50610
H	-2.16280	1.42480	-0.47710
H	-2.23110	1.31680	1.32140
H	-3.78480	3.15310	-0.28150
H	-3.53780	3.31180	1.47840
H	-1.92790	4.89630	2.34760
H	-0.71330	5.57410	1.22900
N	-2.25540	4.49020	0.29910
H	-0.97680	5.33350	-1.15900
H	-2.14210	4.12980	-1.76850
H	1.30990	1.73710	0.30570
H	0.42220	1.18680	1.74750
H	-0.99180	-0.37260	0.33820
O	3.68190	1.38870	-0.18170
H	3.13840	0.59540	0.09760
C	3.81790	1.34840	-1.60150
H	4.53380	2.13710	-1.90810
H	2.85530	1.52960	-2.13330
H	4.22530	0.37170	-1.94390
O	2.68000	3.73670	0.63840
H	3.10410	2.86290	0.38630
C	3.12130	4.71090	-0.29320
H	2.68760	5.69270	-0.01030
H	2.80000	4.49860	-1.34180
H	4.23090	4.82460	-0.29860
O	-3.18830	-0.00160	-3.03390
H	-2.21290	0.24730	-2.64500
C	-3.05290	-0.74580	-4.23020
H	-4.02510	-0.77380	-4.77060
H	-2.73120	-1.80130	-4.06220
H	-2.30390	-0.28060	-4.91250
O	-4.42960	-1.08870	-1.08320
H	-3.95280	-0.64690	-1.88950
C	-4.92850	-2.35150	-1.50020
H	-5.53900	-2.79040	-0.68410
H	-4.12140	-3.08120	-1.74660
H	-5.58410	-2.26150	-2.39620
O	-2.83620	-1.09740	1.03580
H	-3.45680	-1.08360	0.24030
C	-2.71500	-2.44160	1.46520
H	-2.01050	-2.47960	2.32120
H	-2.30790	-3.11880	0.67770
H	-3.68610	-2.86940	1.81330

$TS_{3\text{-}3'}$

C	0.09732	-1.46412	-1.77780
C	-1.00186	-2.34840	-1.75137
C	-0.92768	-3.60901	-2.34577
C	0.27372	-3.99851	-2.95960
C	1.39470	-3.15987	-2.98190
C	1.28816	-1.89041	-2.39848
H	-1.91438	-2.03159	-1.24507
H	-1.76701	-4.30683	-2.33431
N	0.35987	-5.34161	-3.55916
H	2.31566	-3.51223	-3.45142
H	2.14965	-1.21453	-2.42569
C	-0.03392	-0.05223	-1.17441
O	-1.13282	0.62765	-1.58939

H	0.93588	0.47581	-1.46443
O	1.48605	-5.77396	-3.87411
O	-0.70250	-5.98364	-3.68347
C	1.45437	-3.21599	1.82077
O	0.51342	-2.18749	1.42540
C	1.08068	-1.06109	0.91214
O	2.29194	-0.88734	0.81734
C	0.02873	-0.08229	0.42297
C	0.43927	1.27587	1.03644
N	-0.44397	2.51325	0.87265
C	-1.91929	2.21955	1.08003
C	-0.25994	3.15563	-0.50970
C	0.01312	3.53084	1.91425
H	0.84457	-4.10601	2.02161
H	2.18079	-3.40749	1.01918
H	1.99312	-2.90835	2.72990
H	0.79511	3.47342	-0.52955
H	-0.49151	2.35330	-1.24718
C	-1.25411	4.34710	-0.61552
H	1.10270	3.63952	1.79286
C	-0.74662	4.86889	1.66732
H	-0.21892	3.08973	2.89817
C	-2.68303	3.57595	1.15201
H	-2.22002	1.60322	0.22309
H	-2.01453	1.61796	1.99640
H	-3.58288	3.52172	0.52598
H	-3.00761	3.79530	2.18122
H	-1.16829	5.23767	2.61555
H	-0.05574	5.63720	1.28458
N	-1.84042	4.69857	0.69583
H	-0.74021	5.23144	-1.02379
H	-2.07941	4.09456	-1.30073
H	1.40641	1.58781	0.61618
H	0.57274	1.16469	2.12548
H	-0.96312	-0.43853	0.73771
O	3.48889	1.59666	-0.81853
H	2.59610	1.27920	-1.06521
C	4.37554	1.20459	-1.87904
H	5.22498	1.90274	-1.88688
H	3.87730	1.24311	-2.86458
H	4.75770	0.18373	-1.70362
O	2.99249	3.89624	0.59656
H	3.28149	3.09650	0.08473
C	3.28315	5.03742	-0.20541
H	3.18748	5.93142	0.43236
H	2.58758	5.15361	-1.06245
H	4.31520	5.01902	-0.60472
O	-0.76651	1.89119	-4.21363
H	-0.83044	0.92117	-4.06106
C	-1.31131	2.10732	-5.50869
H	-1.44465	3.19475	-5.63373
H	-2.30316	1.62796	-5.63797
H	-0.65350	1.75647	-6.33205
O	-2.47474	-0.39077	-3.68375
H	-2.67531	0.34442	-3.08969
C	-3.29437	-1.23546	-4.45094
H	-4.32667	-0.85388	-4.60347
H	-3.38145	-2.23290	-3.97710
H	-2.84318	-1.35932	-5.44883
O	-3.22911	-0.36241	-0.08469
H	-2.59309	-0.02273	-0.79250
C	-4.30787	-1.04524	-0.71559
H	-4.82569	-1.64040	0.05552
H	-3.96515	-1.73032	-1.51531
H	-5.05077	-0.35433	-1.16699

Intermediate 4

C	7.96522	6.19292	9.34564
C	8.16909	6.45175	7.97479
C	7.17888	6.14553	7.03515
C	5.97731	5.57590	7.48077
C	5.74777	5.29782	8.83531
C	6.75131	5.60635	9.76134
H	9.11037	6.91955	7.65258
H	7.30811	6.34585	5.96320
N	4.91942	5.25987	6.49049
H	4.79293	4.84728	9.13535
H	6.58253	5.40835	10.83070
C	9.00574	6.60692	10.38207

O	10.32964	6.34346	9.97199
H	8.82793	5.99172	11.29203
O	3.86684	4.77642	6.91569
O	5.15847	5.50303	5.30492
C	5.36698	9.27903	9.91376
O	6.77768	8.97595	9.92428
C	7.29494	8.35960	10.99754
O	6.67751	8.02637	11.99733
C	8.79014	8.10362	10.79851
C	9.42676	8.54344	12.12645
N	10.92272	8.80255	12.18056
C	11.40467	9.52230	10.93242
C	11.74102	7.54030	12.35341
C	11.17173	9.69643	13.38993
H	5.29562	10.36037	9.69122
H	4.89809	8.67186	9.11507
H	4.92110	9.03210	10.89558
H	11.44716	7.11675	13.33423
H	11.43221	6.85205	11.54044
C	13.24615	7.93269	12.29391
H	10.70825	9.18194	14.25405
C	12.70437	9.88985	13.55509
H	10.59714	10.62826	13.20292
C	12.85419	10.01350	11.18182
H	11.36521	8.78438	10.11155
H	10.68131	10.33481	10.70949
H	13.48371	9.76912	10.30289
H	12.88359	11.11317	11.31784
H	12.93397	10.96611	13.68664
H	13.08089	9.35836	14.45242
N	13.42391	9.38615	12.38141
H	13.80020	7.44420	13.12054
H	13.69777	7.58513	11.34320
H	9.20106	7.81604	12.92948
H	8.98466	9.52796	12.39381
H	9.10229	8.75389	9.95598
H	10.57620	6.93083	9.18793
O	6.67910	12.12536	9.57455
H	7.66321	11.94035	9.71565
C	6.54332	13.49609	9.27902
H	5.46669	13.72628	9.13431
H	7.07371	13.79815	8.34270
H	6.91508	14.15963	10.09883
O	11.03643	8.05498	8.09486
H	10.40088	8.88247	8.04348
C	11.73848	7.88029	6.87896
H	12.53444	7.12090	7.02491
H	12.22091	8.82616	6.54462
H	11.07843	7.52347	6.05309
O	9.47319	10.02139	8.10961
H	9.41079	10.74103	8.90724
C	8.31748	10.10796	7.28817
H	7.82061	9.11756	7.19596
H	8.57627	10.46652	6.26504
H	7.58206	10.80837	7.73766
O	8.90194	11.53855	12.46873
H	9.04342	11.62420	11.45443
C	7.71864	12.23233	12.82239
H	7.29328	11.78935	13.74850
H	6.95103	12.16453	12.01855
H	7.90814	13.31425	13.02817
C	10.06436	12.82748	9.62065
H	11.16388	12.61477	9.65698
H	9.87576	13.66486	10.33482
H	9.85193	13.21738	8.59564
O	9.29055	11.69389	9.93249

Intermediate 4’

C	-0.00760	-1.45270	-2.04950
C	-1.21190	-2.19070	-2.02580
C	-1.21650	-3.54880	-2.35260
C	-0.00380	-4.16570	-2.70380
C	1.20440	-3.45400	-2.75410
C	1.19030	-2.09230	-2.42970
H	-2.14860	-1.67930	-1.74750
H	-2.14010	-4.14220	-2.33470
N	-0.00150	-5.59590	-3.03410
H	2.13060	-3.96690	-3.04370
H	2.13040	-1.52150	-2.45780

C	0.00000	0.00000	-1.58210
O	-1.01730	0.75880	-2.15750
H	0.97410	0.44760	-1.88600
O	1.07670	-6.12850	-3.32370
O	-1.07780	-6.20460	-3.00500
C	1.90040	-3.02250	1.04120
O	0.83260	-2.08700	0.77650
C	1.18260	-0.85460	0.42050
O	2.36020	-0.48160	0.38320
C	0.00000	0.00000	0.00000
C	0.20570	1.35840	0.69460
N	-0.95600	2.32410	0.77770
C	-2.28800	1.62480	0.95540
C	-1.02580	3.21810	-0.44920
C	-0.71210	3.23110	1.97790
H	1.41020	-3.99620	1.21170
H	2.58580	-3.08310	0.17360
H	2.46990	-2.71530	1.93920
H	-0.09230	3.80950	-0.42690
H	-1.01930	2.54270	-1.32690
C	-2.30490	4.08380	-0.34520
H	0.31800	3.61960	1.86870
C	-1.78230	4.35220	1.97250
H	-0.77230	2.58610	2.87360
C	-3.35870	2.69270	1.29170
H	-2.52750	1.13070	-0.00660
H	-2.16600	0.85630	1.74060
H	-4.26000	2.49750	0.67780
H	-3.66290	2.63770	2.35520
H	-2.20810	4.46370	2.98800
H	-1.32990	5.32440	1.69480
N	-2.86120	4.05100	1.01830
H	-2.07310	5.12930	-0.62830
H	-3.08190	3.72040	-1.04580
H	1.04150	1.92880	0.25060
H	0.47820	1.15570	1.74740
H	-0.91490	-0.51180	0.37580
O	3.43910	1.89000	-0.34190
H	3.03590	1.02780	-0.03730
C	3.45790	1.89340	-1.76880
H	4.07340	2.74930	-2.10970
H	2.44270	2.00250	-2.21540
H	3.91650	0.96490	-2.17340
O	2.15870	4.05860	0.58980
H	2.68880	3.25560	0.30790
C	2.30850	5.04770	-0.41680
H	1.72840	5.94550	-0.11820
H	1.92690	4.72440	-1.41500
H	3.36940	5.36520	-0.54960
O	-2.34990	-1.56810	1.56520
H	-3.18990	-1.47210	1.00440
C	-2.04730	-2.94850	1.63610
H	-1.25080	-3.10770	2.39300
H	-1.67160	-3.36820	0.67220
H	-2.92450	-3.56240	1.95360
O	-4.48400	-1.30390	0.04750
H	-4.01000	-0.66740	-0.66880
C	-4.84550	-2.50270	-0.61300
H	-5.33030	-3.19630	0.10760
H	-3.97510	-3.04500	-1.05750
H	-5.57340	-2.32360	-1.43930
O	-3.34090	0.15120	-1.60790
H	-2.02480	0.42310	-1.89220
C	-4.15970	0.48740	-2.69320
H	-5.22910	0.60760	-2.38870
H	-4.14680	-0.28030	-3.51170
H	-3.86140	1.45690	-3.16720

$TS_{4\text{-}4'}$

C	0.09732	-1.46412	-1.77780
C	-1.00186	-2.34840	-1.75137
C	-0.92768	-3.60901	-2.34577
C	0.27372	-3.99851	-2.95960
C	1.39470	-3.15987	-2.98190
C	1.28816	-1.89041	-2.39848
H	-1.91438	-2.03159	-1.24507
H	-1.76701	-4.30683	-2.33431
N	0.35987	-5.34161	-3.55916
H	2.31566	-3.51223	-3.45142

H	2.14965	-1.21453	-2.42569
C	-0.03392	-0.05223	-1.17441
O	-1.13282	0.62765	-1.58939
H	0.93588	0.47581	-1.46443
O	1.48605	-5.77396	-3.87411
O	-0.70250	-5.98364	-3.68347
C	1.45437	-3.21599	1.82077
O	0.51342	-2.18749	1.42540
C	1.08068	-1.06109	0.91214
O	2.29194	-0.88734	0.81734
C	0.02873	-0.08229	0.42297
C	0.43927	1.27587	1.03644
N	-0.44397	2.51325	0.87265
C	-1.91929	2.21955	1.08003
C	-0.25994	3.15563	-0.50970
C	0.01312	3.53084	1.91425
H	0.84457	-4.10601	2.02161
H	2.18079	-3.40749	1.01918
H	1.99312	-2.90835	2.72990
H	0.79511	3.47342	-0.52955
H	-0.49151	2.35330	-1.24718
C	-1.25411	4.34710	-0.61552
H	1.10270	3.63952	1.79286
C	-0.74662	4.86889	1.66732
H	-0.21892	3.08973	2.89817
C	-2.68303	3.57595	1.15201
H	-2.22002	1.60322	0.22309
H	-2.01453	1.61796	1.99640
H	-3.58288	3.52172	0.52598
H	-3.00761	3.79530	2.18122
H	-1.16829	5.23767	2.61555
H	-0.05574	5.63720	1.28458
N	-1.84042	4.69857	0.69583
H	-0.74021	5.23144	-1.02379
H	-2.07941	4.09456	-1.30073
H	1.40641	1.58781	0.61618
H	0.57274	1.16469	2.12548
H	-0.96312	-0.43853	0.73771
O	3.48889	1.59666	-0.81853
H	2.59610	1.27920	-1.06521
C	4.37554	1.20459	-1.87904
H	5.22498	1.90274	-1.88688
H	3.87730	1.24311	-2.86458
H	4.75770	0.18373	-1.70362
O	2.99249	3.89624	0.59656
H	3.28149	3.09650	0.08473
C	3.28315	5.03742	-0.20541
H	3.18748	5.93142	0.43236
H	2.58758	5.15361	-1.06245
H	4.31520	5.01902	-0.60472
O	-0.76651	1.89119	-4.21363
H	-0.83044	0.92117	-4.06106
C	-1.31131	2.10732	-5.50869
H	-1.44465	3.19475	-5.63373
H	-2.30316	1.62796	-5.63797
H	-0.65350	1.75647	-6.33205
O	-2.47474	-0.39077	-3.68375
H	-2.67531	0.34442	-3.08969
C	-3.29437	-1.23546	-4.45094
H	-4.32667	-0.85388	-4.60347
H	-3.38145	-2.23290	-3.97710
H	-2.84318	-1.35932	-5.44883
O	-3.22911	-0.36241	-0.08469
H	-2.59309	-0.02273	-0.79250
C	-4.30787	-1.04524	-0.71559
H	-4.82569	-1.64040	0.05552
H	-3.96515	-1.73032	-1.51531
H	-5.05077	-0.35433	-1.16699

$TS_{4'-5}$

C	10.99397	11.71387	4.90073
C	12.27738	11.62949	4.31838
C	12.44001	11.17267	3.00862
C	11.30643	10.80233	2.27032
C	10.01891	10.86167	2.82054
C	9.87894	11.31184	4.13854
H	13.15977	11.93085	4.88872
H	13.42231	11.10568	2.53624
N	11.47317	10.34527	0.87699

H	9.16441	10.55964	2.21275
H	8.88076	11.36000	4.58672
C	10.80169	12.17861	6.34277
O	11.38341	11.23724	7.25683
H	9.71231	12.08041	6.56204
O	10.44583	10.06152	0.23314
O	12.63207	10.27116	0.42688
C	9.98108	15.21775	3.50545
O	10.80801	14.47568	4.42542
C	10.47390	14.59140	5.75579
O	9.62372	15.45457	6.09577
C	11.20229	13.66134	6.62910
C	10.89105	14.10044	8.05257
N	11.85667	13.74203	9.23086
C	13.26725	13.38506	8.80553
C	11.27781	12.58343	10.03700
C	11.93460	14.95142	10.15693
H	10.50997	15.18885	2.54369
H	8.99015	14.74498	3.40240
H	9.84576	16.25460	3.84538
H	10.32442	12.96278	10.43948
H	11.10136	11.77849	9.30733
C	12.29489	12.19716	11.15045
H	10.89673	15.27332	10.34754
C	12.67965	14.53663	11.46023
H	12.47386	15.72706	9.59113
C	14.15133	13.25839	10.08088
H	13.19123	12.43281	8.26661
H	13.61235	14.15987	8.10575
H	14.74480	12.33266	10.01648
H	14.85571	14.10256	10.16083
H	13.43842	15.29255	11.71882
H	11.97062	14.46981	12.30267
N	13.33993	13.22567	11.31309
H	11.76854	12.06934	12.11119
H	12.79284	11.24307	10.91251
H	9.90793	13.76385	8.43002
H	10.86253	15.19933	8.05714
H	12.80853	14.10604	6.14483
O	13.97461	10.53635	7.17635
H	12.32919	11.01474	7.01796
C	14.37126	9.18422	6.96673
H	15.27928	8.92666	7.54987
H	14.56642	8.96176	5.89978
H	13.55007	8.53353	7.30489
O	15.35608	12.33669	5.87797
H	14.60875	11.15556	6.69691
C	16.29017	12.25003	4.80716
H	16.67685	13.24480	4.52360
H	15.85422	11.77132	3.90802
H	17.14500	11.63456	5.13361
O	13.81963	14.42039	5.97556
H	14.79930	13.19238	5.83506
C	13.88268	15.42128	4.96044
H	13.18457	16.25190	5.17289
H	13.64241	15.02286	3.95746
H	14.90556	15.83181	4.94281
O	8.82803	15.03416	10.55990
H	8.39405	14.99255	9.65997
C	7.83574	15.37027	11.52296
H	8.34673	15.57093	12.47967
H	7.10977	14.54949	11.69751
H	7.26421	16.27836	11.24581
O	7.77253	15.02303	8.05974
H	8.45364	15.19926	7.34571
C	6.93997	13.94535	7.63996
H	6.20023	13.76578	8.43593
H	7.50492	13.00462	7.47735
H	6.39115	14.18598	6.71014

TS_{4-5}

C	7.87508	6.62857	9.31059
C	8.10476	6.59486	7.92255
C	7.11523	6.13376	7.04485
C	5.89158	5.69182	7.56724
C	5.64095	5.68460	8.94471
C	6.63982	6.14635	9.80500
H	9.06757	6.91898	7.52058
H	7.27064	6.10384	5.96583

N	4.84728	5.22368	6.63837
H	4.68225	5.31557	9.31360
H	6.45853	6.14341	10.88555
C	8.94160	7.08063	10.31325
O	10.25434	6.66509	9.92735
H	8.74547	6.45232	11.20469
O	3.76728	4.84541	7.12863
O	5.10362	5.24110	5.42257
C	5.30834	9.38802	10.10007
O	6.74585	9.24181	10.01697
C	7.38880	8.88432	11.15256
O	6.84406	8.81682	12.25208
C	8.85910	8.56959	10.87560
C	9.51870	8.59458	12.26857
N	11.05787	8.78871	12.33152
C	11.55794	9.46435	11.07501
C	11.83682	7.49049	12.49176
C	11.35870	9.67143	13.53318
H	5.08258	10.30646	9.58256
H	4.83823	8.52237	9.60620
H	4.99997	9.44155	11.15354
H	11.56648	7.07752	13.47935
H	11.48425	6.82086	11.69193
C	13.35780	7.83981	12.37481
H	10.87093	9.19732	14.40087
C	12.90410	9.79454	13.69478
H	10.85052	10.63305	13.34150
C	13.00516	9.96174	11.31232
H	11.48509	8.70991	10.27791
H	10.78614	10.20413	10.88423
H	13.62602	9.74630	10.42665
H	13.03372	11.05035	11.48479
H	13.17617	10.85198	13.85367
H	13.26551	9.22318	14.56701
N	13.60338	9.29562	12.49453
H	13.93153	7.31625	13.15389
H	13.75117	7.51182	11.39880
H	9.34857	7.68114	12.86016
H	9.11086	9.44196	12.81315
H	9.10384	9.76796	10.19511
H	10.53566	7.09276	9.08298
O	6.51330	12.06065	9.09909
H	7.42117	11.77262	9.39932
C	6.43174	13.47650	9.17597
H	5.39321	13.75525	8.93541
H	7.09980	13.99537	8.46077
H	6.64929	13.86698	10.19215
O	11.05579	8.05310	7.76898
H	10.40482	8.80858	7.59083
C	12.11353	8.05792	6.81594
H	12.81070	7.24300	7.07935
H	12.67147	9.01263	6.82119
H	11.75674	7.87597	5.78310
O	9.36408	9.99395	7.47054
H	9.14000	10.32941	8.38609
C	8.15121	9.96953	6.69355
H	8.44085	9.90300	5.63608
H	7.56777	10.89269	6.86692
H	7.52454	9.10884	6.95509
O	8.69161	11.52412	12.60761
H	8.78885	11.44203	11.61638
C	7.33937	11.91449	12.86868
H	6.85694	11.17841	13.53174
H	6.75125	11.96048	11.93450
H	7.30800	12.90941	13.35439
C	10.09185	12.14426	9.54380
H	11.11088	11.70770	9.62033
H	10.05906	13.03187	10.20622
H	9.96910	12.48334	8.49707
O	9.05750	11.22526	9.90953

Intermediate 5

C	14.09486	14.90315	8.40272
C	13.94083	13.97230	7.36323
C	12.67990	13.40427	7.15139
C	11.57965	13.76176	7.95837
C	11.76664	14.70168	8.99544
C	13.01885	15.27660	9.22684
C	10.20766	13.11861	7.77141

O	10.17227	12.38430	6.51481
N	15.41088	15.50198	8.63467
O	15.52664	16.32751	9.54969
O	16.35089	15.15498	7.90731
C	9.04923	14.07114	7.90533
C	8.01287	13.87557	8.92368
N	6.88533	12.79574	8.55275
C	7.39219	11.40717	8.83390
C	6.30483	10.37847	8.42001
C	8.89365	15.08105	6.93717
O	9.86962	15.01052	5.93637
C	9.89462	16.07542	4.98523
C	5.65994	13.05692	9.38721
C	4.62190	11.93244	9.11480
C	6.50221	12.88402	7.09996
C	5.34618	11.88115	6.83093
H	7.42627	14.79554	9.10010
H	10.69267	15.82379	4.26227
H	8.92480	16.17233	4.45562
H	10.13233	17.04529	5.47022
H	8.40950	13.48356	9.88014
H	10.12333	12.35088	8.56963
H	10.90598	14.99165	9.61459
H	13.17678	16.00925	10.02942
H	14.80300	13.70548	6.73817
H	12.53640	12.67872	6.33898
H	8.32107	11.27128	8.25836
H	7.63878	11.36680	9.90994
H	6.20982	13.93365	6.92673
H	7.41839	12.66796	6.52061
H	5.98546	13.09343	10.44351
H	5.29948	14.05905	9.08454
N	5.06308	11.05620	8.01686
H	6.66913	9.76045	7.57797
H	6.08854	9.69358	9.26222
H	4.47137	11.31077	10.01830
H	3.64277	12.37752	8.85186
H	4.42122	12.42208	6.54903
H	5.60662	11.21514	5.98558
O	8.01680	15.98586	6.84493
H	10.08883	13.08699	5.82544
O	10.24330	9.85088	5.91956
H	10.22813	10.83639	6.13545
C	11.58849	9.48448	5.61894
H	11.61767	8.39578	5.41312
H	12.29510	9.69585	6.45233
H	11.95604	10.01328	4.71193
O	5.54565	15.96404	7.70142
H	6.52397	15.98715	7.44878
C	4.81444	16.23260	6.51874
H	3.73012	16.24449	6.75966
H	4.96894	15.46194	5.72343
H	5.06891	17.22341	6.07169
O	9.15293	8.92445	8.08445
H	9.57762	9.20450	7.20162
C	8.93305	7.51899	8.09191
H	8.42275	7.24600	9.03782
H	9.88295	6.93951	8.03501
H	8.28186	7.20430	7.24630
O	9.81672	12.21831	11.03168
H	10.08110	11.34457	10.60856
C	10.93871	12.67385	11.76703
H	10.68230	13.63985	12.25061
H	11.83682	12.85327	11.12986
H	11.23677	11.96601	12.57791
O	10.64240	9.94919	9.93957
H	10.02602	9.52281	9.24805
C	11.92667	10.08279	9.34225
H	12.66196	10.36303	10.12389
H	11.95818	10.87090	8.55361
H	12.27031	9.12446	8.89281

Intermediate 6

C	9.32898	6.11658	8.44461
C	10.10074	7.04212	7.72808
C	9.57051	8.26002	7.27567
C	8.24593	8.57388	7.58896
C	7.46062	7.67780	8.34467
C	8.00245	6.44132	8.74511

C	6.03141	8.06657	8.72471
O	5.34746	7.04931	9.44227
C	6.03234	9.38880	9.49477
C	6.54392	9.29008	10.88702
O	6.92353	8.21045	11.37742
C	5.62108	10.55913	8.96259
O	6.54122	10.42939	11.57378
C	6.89865	10.35957	12.97737
H	5.27324	10.61973	7.92080
H	5.63663	11.48950	9.54919
H	6.67977	11.36344	13.38158
H	6.27338	9.60082	13.48582
H	7.97459	10.11471	13.11359
H	5.46073	8.22803	7.78655
H	5.78447	7.02459	10.32631
N	11.51764	6.75709	7.50040
H	10.20575	8.95040	6.70755
H	7.81831	9.53240	7.25550
H	7.37371	5.73181	9.30042
H	9.78070	5.17291	8.77530
O	11.98681	5.70549	7.95311
O	12.19227	7.58681	6.87605
O	9.45091	7.25821	11.73132
H	8.54915	7.66454	11.64710
C	9.30386	5.90346	12.14522
H	10.31418	5.45562	12.22034
H	8.70773	5.29972	11.42523
H	8.82013	5.83607	13.14414
O	10.97874	10.48352	9.12848
H	11.08093	9.55329	9.50985
C	12.13203	10.79795	8.36175
H	11.90373	11.68437	7.73459
H	12.43148	9.96543	7.68784
H	13.00514	11.05566	9.00538
O	11.32124	8.07328	10.09580
H	10.56007	7.70969	10.65406
C	12.55291	7.62226	10.63712
H	13.38015	8.08379	10.06199
H	12.65671	6.51734	10.54973
H	12.68131	7.89933	11.70785
O	8.88873	11.84603	8.40943
H	9.63388	11.21265	8.67128
C	8.53704	12.62529	9.53977
H	7.78400	13.37945	9.23008
H	9.41012	13.18194	9.95104
H	8.08846	12.01935	10.35982
O	7.23221	11.49503	6.40016
H	7.81105	11.63087	7.21160
C	8.09547	11.57237	5.27992
H	7.48851	11.51230	4.35196
H	8.83914	10.73838	5.23823
H	8.66997	12.52858	5.24266
N	12.52845	11.23127	13.44324
C	11.89480	11.16197	12.10930
C	11.72892	12.12926	14.30174
C	12.52596	9.87867	14.04039
H	12.22609	12.20204	15.29064
H	11.74077	13.14336	13.85059
C	10.26952	11.58539	14.43898
H	12.49976	10.48859	11.47069
C	10.42854	10.64307	12.23637
H	11.93178	12.17096	11.64924
H	13.00388	9.93951	15.04000
C	11.06522	9.33123	14.13705
H	13.16573	9.22078	13.41480
H	10.02042	11.35703	15.49615
H	9.52456	12.32412	14.07553
H	9.70016	11.39035	11.86342
H	10.28897	9.71572	11.65269
N	10.11782	10.35033	13.64779
H	10.91952	8.42136	13.51984
H	10.79813	9.07313	15.18310

Clusters with 10 methanol molecules (labels follow main text)

Intermediate 1

C	10.03130	13.28400	13.63890
C	10.85767	12.66802	14.60420
C	10.88281	11.27625	14.71664

C	10.09122	10.51931	13.83766	H	11.81020	12.18781	12.02556
C	9.26070	11.10400	12.86892	C	13.92133	8.10738	14.30106
C	9.23200	12.49977	12.77913	H	14.73104	10.08598	14.53649
H	11.47959	13.29239	15.25699	H	14.48580	7.65352	15.12993
H	11.51059	10.76750	15.45950	H	13.22687	7.46467	13.73651
N	10.14209	9.04723	13.93245	O	16.77510	10.52349	12.53705
H	8.67373	10.46657	12.19676	H	16.37675	11.15223	13.22707
H	8.59966	12.98717	12.02181	C	17.49606	9.48750	13.19192
C	10.01206	14.76387	13.50743	H	17.98950	8.86065	12.42111
O	10.72293	15.51910	14.16466	H	16.84389	8.82322	13.80506
H	9.24961	15.16477	12.78861	H	18.29368	9.89373	13.85370
O	9.55274	8.38981	13.07070	O	15.97332	14.72463	11.36755
O	10.77765	8.55137	14.86773	H	15.02309	14.92452	11.57641
N	12.92087	15.44069	7.72135	C	16.06149	13.32470	11.12993
C	13.83536	14.72384	8.63286	H	17.13173	13.04438	11.06147
C	13.20977	16.88538	7.79963	H	15.56780	13.00714	10.18219
C	13.15482	14.97855	6.34020	H	15.62014	12.73678	11.95993
H	12.51045	17.42125	7.12486	O	10.47770	12.55548	9.65117
H	12.99415	17.22712	8.83380	H	10.52720	11.58529	9.93801
C	14.70041	17.15535	7.41112	C	10.96667	12.63561	8.30658
H	13.59525	13.64228	8.62647	H	11.13500	13.70395	8.06323
C	15.31543	14.97340	8.20171	H	10.22598	12.22449	7.58406
H	13.65236	15.09643	9.65949	H	11.93017	12.09564	8.19043
H	12.43384	15.49228	5.67117	O	15.06532	9.82166	10.68963
C	14.63147	15.27301	5.92072	H	15.74965	10.10416	11.38314
H	12.92888	13.89291	6.29457	C	15.13428	8.42172	10.49197
H	14.77217	17.85395	6.55098	H	14.28608	8.11007	9.84907
H	15.26183	17.61099	8.25300	H	15.05219	7.85950	11.44968
H	15.89781	15.40633	9.04061	H	16.07807	8.10430	9.98763
H	15.81172	14.02440	7.90916	O	13.39449	15.27994	12.16139
N	15.36741	15.88985	7.04332	H	12.53346	14.98539	11.68628
H	15.16211	14.34397	5.62482	C	13.30915	16.68852	12.41280
H	14.67423	15.96421	5.05292	H	14.22101	17.00461	12.95739
C	13.26844	10.00629	12.88813	H	12.42162	16.93347	13.03264
O	12.40488	9.39906	12.24322	H	13.26226	17.26337	11.46245
O	13.56004	11.29904	12.71418	O	15.94310	12.22216	14.36120
C	14.05806	9.41025	13.98709	H	15.05574	12.70855	14.29561
C	12.84102	11.99093	11.68412	C	16.94379	13.23259	14.48429
H	13.36842	12.94129	11.52679	H	17.93625	12.77640	14.29143
H	12.83715	11.41209	10.74640	H	16.96688	13.66418	15.51043

H	16.79364	14.05803	13.75357
O	13.84240	13.80219	14.35122
H	13.64573	14.34017	13.52732
C	13.85847	14.69061	15.46218
H	13.99904	14.09213	16.38502
H	12.89828	15.24339	15.54986
H	14.68701	15.43503	15.41036
O	10.71017	9.98830	10.26192
H	11.28486	9.84448	11.06747
C	11.33002	9.25591	9.19896
H	10.69725	9.35455	8.29451
H	11.39966	8.17380	9.44789
H	12.34494	9.64555	8.96310
O	11.16629	14.63253	11.02428
H	10.97316	13.75161	10.54166
C	10.53664	15.67652	10.28133
H	10.57630	16.61314	10.87310
H	9.46732	15.44055	10.08855
H	11.04219	15.84287	9.30420
O	14.13730	11.28971	8.70238
H	14.49864	10.77193	9.48475
C	14.95203	10.97145	7.59210
H	14.65214	11.60575	6.73136
H	14.84733	9.90781	7.26472
H	16.03565	11.15534	7.78316

Intermediate 2

C	15.72129	12.50966	10.37372
C	16.83221	13.07279	11.03839
C	16.75336	14.36727	11.55751
C	15.56627	15.08972	11.36371
C	14.47619	14.58002	10.64789
C	14.55379	13.27327	10.17165
H	17.73395	12.46235	11.19453
H	17.58233	14.81626	12.11908
N	15.44989	16.43173	11.94589
H	13.58711	15.19618	10.48049
H	13.70502	12.82838	9.64067
C	15.71590	11.07590	9.98784
O	16.72662	10.38291	9.86570
H	14.69189	10.64024	9.91262
O	14.43936	17.09449	11.68299
O	16.36762	16.83945	12.66900
C	10.90743	12.70301	12.55489
O	10.53889	11.64845	11.64345
C	11.54683	11.04952	10.97251
O	12.74233	11.23503	11.25971
C	11.08574	10.18245	9.89417
C	11.96806	9.68782	8.98002
N	13.01725	7.63882	9.83347
C	11.95804	7.09720	10.69396
C	14.18242	8.00952	10.65492
C	13.41775	6.63365	8.83842
H	9.96322	13.07128	12.99196
H	11.57194	12.32934	13.35846
H	11.40712	13.51909	11.99824
H	14.96488	8.43294	9.99366
H	13.86562	8.80839	11.35459
C	14.71160	6.73941	11.40141
H	14.19238	7.08159	8.18240
C	13.95554	5.36395	9.58451
H	12.53913	6.39285	8.20575
C	12.49432	5.82982	11.43638
H	11.64526	7.89435	11.40054
H	11.08031	6.85953	10.05875
H	12.45768	5.96173	12.53710
H	11.88499	4.93640	11.19014
H	13.35672	4.46659	9.32698
H	15.00732	5.15129	9.30428
N	13.89394	5.56314	11.04646
H	15.76708	6.52992	11.13231
H	14.67206	6.87420	12.50184
H	13.00909	10.03529	8.96822
H	11.61974	9.10267	8.11751
H	10.01088	9.95094	9.85368
O	14.75221	11.37508	7.56007
H	15.49432	12.07780	7.43009
C	15.11650	10.19489	6.85529
H	14.23364	9.52797	6.78825

H	15.93043	9.64647	7.38215
H	15.45432	10.41656	5.81860
O	11.24551	13.58678	9.37030
H	11.74870	13.13833	8.60692
C	9.84953	13.47404	9.10690
H	9.28862	13.81290	10.00054
H	9.56036	12.42181	8.89905
H	9.53329	14.09928	8.24024
O	16.61877	13.08800	7.16851
H	16.48085	13.98539	7.63126
C	17.92379	12.61222	7.48697
H	18.08795	11.64830	6.96429
H	18.06662	12.43790	8.57626
H	18.70613	13.32113	7.13576
O	14.10065	12.14916	13.31007
H	13.54808	11.86848	12.51843
C	15.06060	11.14322	13.58874
H	15.70946	11.49457	14.41632
H	15.71863	10.91905	12.71924
H	14.58007	10.19284	13.91472
O	11.88021	16.11211	9.35814
H	11.66092	15.11681	9.34745
C	11.36072	16.66101	10.57151
H	11.68843	17.71655	10.64768
H	11.71201	16.11622	11.47565
H	10.24659	16.65188	10.56875
O	14.27540	14.70598	13.80683
H	14.27345	13.72775	13.54906
C	15.01450	14.85181	15.00670
H	14.98030	15.91536	15.31911
H	16.08568	14.57524	14.87024
H	14.60421	14.23610	15.84114
O	14.15814	16.84952	8.26080
H	13.31098	16.54433	8.72460
C	14.37569	18.21911	8.59023
H	15.23849	18.59195	8.00149
H	14.59817	18.36950	9.67007
H	13.49442	18.84509	8.32406
O	12.52964	12.70521	7.29063
H	13.32390	12.08180	7.39565
C	13.03795	13.90518	6.70557
H	12.19296	14.60008	6.52924
H	13.52540	13.70437	5.72652
H	13.77631	14.42085	7.35789
O	11.77685	15.60357	13.79636
H	12.69219	15.19361	13.80292
C	11.97403	16.97420	14.09277
H	11.05248	17.54046	13.83408
H	12.81676	17.42570	13.51857
H	12.17167	17.15847	15.17926
O	16.32073	15.37195	8.34733
H	15.47148	15.92727	8.32162
C	17.42384	16.18069	8.71423
H	18.30087	15.52764	8.89816
H	17.23577	16.74765	9.65400
H	17.70473	16.91306	7.92144

Intermediate 3

C	12.82840	13.34814	8.73343
C	11.70820	12.83280	8.04555
C	10.41404	13.06554	8.51222
C	10.24708	13.82416	9.68433
C	11.33776	14.35333	10.38886
C	12.62756	14.10684	9.90225
H	11.85165	12.25914	7.12194
H	9.53372	12.67671	7.98514
N	8.88911	14.06514	10.18902
H	11.16920	14.93486	11.30416
H	13.49537	14.50925	10.44113
C	14.24004	13.09986	8.21432
O	14.42237	11.74555	7.87539
H	14.93861	13.39561	9.03040
O	8.75792	14.74373	11.21435
O	7.93976	13.58011	9.56199
C	11.68588	16.38051	6.90756
O	12.57355	15.28036	6.63084
C	13.80431	15.36289	7.16161
O	14.21933	16.35131	7.75491
C	14.58144	14.06007	7.00288

C	16.04252	14.51056	6.86330	H	17.71473	15.58003	9.43101
N	17.10886	13.52031	6.45988	C	17.77334	17.29983	8.47977
C	16.73489	12.74591	5.21975	H	18.41979	17.78967	7.72139
C	17.41580	12.53710	7.56421	H	17.65065	18.01848	9.32550
C	18.36197	14.34123	6.16036	H	16.76850	17.14448	8.02307
H	10.70989	16.10211	6.47403	O	13.57889	10.99125	5.65901
H	11.58950	16.53696	8.00042	H	13.90638	11.33175	6.62840
H	12.05770	17.31458	6.44364	C	12.46495	10.09327	5.76017
H	17.61135	13.13395	8.47180	H	12.67401	9.16207	5.19184
H	16.49128	11.95595	7.71073	H	11.54694	10.55145	5.33303
C	18.61269	11.65041	7.12882	H	12.26781	9.82252	6.81791
H	18.57380	14.90763	7.09076	O	14.30678	15.23984	4.06178
C	19.50100	13.37973	5.74058	H	14.13769	14.25169	3.92393
H	18.06565	15.05456	5.36744	C	13.17930	15.94797	3.56311
C	17.92377	11.81920	4.83816	H	13.25188	17.00753	3.88269
H	15.83165	12.16230	5.46317	H	12.22782	15.53413	3.96122
H	16.48898	13.49126	4.44356	H	13.12806	15.93142	2.44943
H	17.57180	10.76945	4.85896	O	15.98942	10.76999	9.70860
H	18.27047	12.04731	3.81173	H	15.33918	11.04950	8.97242
H	19.85155	13.62230	4.71933	C	15.85811	9.39505	10.05557
H	20.36584	13.48644	6.42340	H	16.62878	8.77144	9.54801
N	19.05136	11.97948	5.76590	H	14.85223	9.01194	9.78911
H	19.47038	11.78460	7.81676	H	15.99895	9.27837	11.15089
H	18.32645	10.58190	7.16838	O	15.71632	9.42243	5.23061
H	16.38898	14.95120	7.81297	H	14.92655	10.03396	5.26687
H	16.08961	15.27848	6.07020	C	15.92072	8.97780	6.56235
H	14.21269	13.58036	6.07992	H	16.74663	8.23657	6.56574
O	13.72023	12.71420	3.69988	H	15.02052	8.47594	6.98947
H	13.56714	12.08284	4.47795	H	16.19857	9.80132	7.25785
C	14.41724	11.99875	2.68387	O	16.65773	16.41825	4.21885
H	14.72051	12.71465	1.89383	H	15.78564	15.95244	4.03964
H	13.77213	11.22259	2.21438	C	16.34535	17.65490	4.84357
H	15.33516	11.49365	3.06019	H	17.28784	18.12726	5.18887
O	16.96556	12.55624	11.27959	H	15.68211	17.53782	5.73297
H	16.50348	11.85665	10.69699	H	15.85183	18.37144	4.14426
C	18.35066	12.22100	11.31505	O	12.78229	10.02238	9.16236
H	18.87336	12.93545	11.98277	H	13.33771	10.71433	8.69627
H	18.83718	12.27133	10.31352	C	12.49972	10.49448	10.46947
H	18.50940	11.19606	11.71773	H	12.19249	9.63405	11.10140
O	18.39135	16.08575	8.88555	H	11.66519	11.23635	10.49108

H	13.38545	10.96518	10.95521
O	16.44123	14.83513	10.16893
H	16.67456	13.97102	10.66106
C	15.79319	15.73893	11.05773
H	15.17731	16.44449	10.46455
H	16.51959	16.32838	11.66354
H	15.12806	15.20569	11.77089

Intermediate 4

C	15.47190	13.79801	16.21999
C	14.45322	13.63656	17.18450
C	14.15338	14.66858	18.07700
C	14.88708	15.86461	18.00125
C	15.92126	16.04409	17.07024
C	16.20857	14.99978	16.18347
H	13.88669	12.69369	17.23287
H	13.35717	14.56454	18.82601
N	14.57015	16.95611	18.93102
H	16.48303	16.98674	17.05113
H	17.01521	15.12747	15.44566
C	15.72588	12.71341	15.18247
O	15.79608	11.43337	15.77183
H	16.72094	12.90958	14.72671
O	15.21680	18.00680	18.84185
O	13.66747	16.77672	19.75732
C	13.37232	16.24938	14.34412
O	13.48667	14.81831	14.40959
C	14.46782	14.26391	13.67889
O	15.18127	14.90003	12.91248
C	14.66476	12.78968	14.02153
C	15.04866	12.10918	12.70042
N	15.19384	10.60428	12.64396
C	14.07114	9.90471	13.37228
C	16.53926	10.13792	13.17446
C	15.11731	10.20931	11.17569
H	12.56249	16.52239	15.04275
H	14.32079	16.73076	14.65562
H	13.12145	16.58605	13.31965
H	17.29193	10.65133	12.54571
H	16.60642	10.49302	14.21739
C	16.59317	8.59017	13.08935
H	15.89119	10.80059	10.65447
C	15.33999	8.67854	11.05808
H	14.10966	10.50488	10.82107
C	14.20949	8.37483	13.14661
H	14.14987	10.18957	14.43344
H	13.13200	10.30541	12.95657
H	14.24432	7.85025	14.12023
H	13.32184	7.99626	12.60664
H	14.50383	8.22262	10.49401
H	16.27265	8.45913	10.50306
N	15.42251	8.04810	12.38351
H	17.51421	8.26946	12.56412
H	16.62262	8.14785	14.10398
H	15.99560	12.50896	12.29677
H	14.25818	12.34144	11.96712
H	13.69185	12.41996	14.41875
H	14.89744	11.14023	16.14900
O	13.81017	13.57068	9.88500
H	13.20491	13.51690	10.69830
C	13.12227	13.00793	8.77113
H	13.86517	12.78355	7.97756
H	12.38011	13.72353	8.34384
H	12.59652	12.06846	9.04694
O	16.38866	13.72486	9.78959
H	15.39113	13.56263	9.89438
C	16.55849	15.13962	9.82757
H	17.64063	15.37127	9.75054
H	16.16664	15.58513	10.76741
H	16.04986	15.63441	8.96851
O	11.09326	8.98815	13.52421
H	10.95941	9.94291	13.15945
C	9.92437	8.20459	13.32686
H	10.14213	7.16011	13.63312
H	9.61358	8.18455	12.25931
H	9.05706	8.56082	13.92935
O	12.01083	10.44663	10.45153
H	11.52918	10.80805	11.26741
C	11.54169	9.13901	10.18971

H	12.02034	8.76816	9.25905
H	10.43827	9.10585	10.01915
H	11.77846	8.41461	11.00357
O	11.77240	11.90297	15.06251
H	11.40174	11.71758	14.14522
C	11.05158	12.98886	15.60349
H	11.50998	13.27773	16.57151
H	9.98236	12.73790	15.81167
H	11.06986	13.89268	14.95239
O	12.23261	13.47981	11.93338
H	11.69747	12.64555	12.21354
C	11.37397	14.60820	11.88790
H	11.94485	15.46864	11.48292
H	10.99736	14.89033	12.89616
H	10.49749	14.44911	11.21951
O	13.69967	10.44657	16.92692
H	12.90312	10.02853	16.44345
C	14.26737	9.43593	17.75539
H	15.11737	9.87742	18.31416
H	14.65500	8.57168	17.16985
H	13.53392	9.05080	18.49777
O	18.04880	12.50055	11.44209
H	17.42929	12.94672	10.78133
C	18.45608	13.48728	12.37708
H	18.96269	12.98748	13.22970
H	17.59840	14.07490	12.77946
H	19.18624	14.21371	11.94424
O	11.74078	9.00603	16.04065
H	11.55325	8.99420	15.03990
C	10.54072	9.44772	16.66810
H	10.78244	9.82519	17.68319
H	9.80753	8.61584	16.78155
H	10.06084	10.27770	16.10709
C	9.48981	11.70550	12.43343
H	9.22590	12.69571	12.87206
H	8.82057	10.95841	12.92136
H	9.20807	11.73900	11.35394
O	10.86063	11.38928	12.61396

Intermediate 5

C	9.62511	14.90619	16.10695
C	10.98990	14.81649	15.79279
C	11.48115	13.60540	15.30312
C	10.63049	12.49817	15.12622
C	9.25984	12.61979	15.44046
C	8.74517	13.82324	15.92390
C	11.16168	11.17027	14.63111
O	12.50893	10.95579	15.19921
N	9.11831	16.14357	16.69384
O	7.90466	16.22767	16.93572
O	9.92589	17.04990	16.94231
C	11.03017	10.90984	13.14915
C	11.06185	9.48134	12.73156
N	12.31944	8.99647	11.94054
C	13.56411	9.08297	12.78013
C	14.78056	8.62518	11.93086
C	10.43981	11.79231	12.21745
O	10.36431	13.14178	12.60162
C	9.31523	13.91235	11.99542
C	12.08608	7.55658	11.53917
C	13.36989	7.01759	10.85217
C	12.53158	9.80768	10.68462
C	13.70135	9.18160	9.87746
H	10.23853	9.21952	12.04034
H	9.38334	14.92302	12.43774
H	9.43490	13.97189	10.89545
H	8.32304	13.47705	12.23242
H	11.02196	8.78427	13.59209
H	10.57755	10.39579	15.17523
H	8.59377	11.75494	15.30605
H	7.68545	13.93205	16.18824
H	11.65090	15.68255	15.91327
H	12.54914	13.52837	15.06280
H	13.64308	10.13315	13.10716
H	13.40534	8.44520	13.66632
H	11.57116	9.78607	10.14517
H	12.73399	10.84290	11.01396
H	11.81324	7.00846	12.46087
H	11.20945	7.57195	10.86324

N	14.34856	8.09503	10.62911
H	15.47065	9.47192	11.74791
H	15.34729	7.84375	12.47317
H	13.84491	6.23589	11.47559
H	13.11249	6.55604	9.87954
H	13.32902	8.77435	8.91840
H	14.45831	9.95330	9.63790
O	9.96781	11.51341	11.08079
H	13.27911	11.41448	14.68920
O	12.51030	14.64431	12.26016
H	11.72812	14.06533	12.49765
C	13.48801	13.81132	11.63325
H	14.31842	14.45461	11.28002
H	13.06124	13.30043	10.74177
H	13.91071	13.04888	12.32436
O	9.39892	9.28408	9.80169
H	9.56468	10.11150	10.35033
C	9.29478	9.69765	8.44934
H	9.09828	8.80732	7.81587
H	10.22960	10.17487	8.06522
H	8.45858	10.41889	8.28398
O	13.50132	15.99014	14.24657
H	13.04712	15.49772	13.47989
C	13.96532	17.25263	13.77818
H	14.50033	17.76013	14.60602
H	13.11721	17.90204	13.46785
H	14.66761	17.15673	12.91876
O	14.68548	11.89650	14.27839
H	14.85968	12.85059	14.62015
C	15.67652	11.02833	14.83676
H	15.20550	10.07465	15.15557
H	16.16413	11.48119	15.72572
H	16.47184	10.79716	14.09545
O	10.26248	10.21208	17.63587
H	11.04582	10.80779	17.77901
C	9.18616	10.73020	18.39661
H	8.27523	10.14175	18.16071
H	8.96376	11.79634	18.16030
H	9.35697	10.65338	19.49834
O	11.90067	14.31801	18.58018
H	12.00845	13.44563	18.11490
C	10.67860	14.26989	19.28949
H	10.54712	15.22785	19.83531
H	10.64704	13.44976	20.04718
H	9.78884	14.14117	18.62756
O	11.07984	6.98925	14.85853
H	11.87369	7.47042	15.23614
C	10.00012	7.20449	15.76047
H	9.04860	6.95763	15.24382
H	9.93546	8.25662	16.12285
H	10.06839	6.55002	16.66304
O	12.44996	11.82471	17.69409
H	12.56213	11.54434	16.72750
C	13.62697	11.53767	18.45760
H	13.83073	12.39588	19.12966
H	14.51019	11.38163	17.80471
H	13.48734	10.63309	19.08514
O	15.10776	14.24785	15.22052
H	14.52366	14.96972	14.78088
C	14.96810	14.40067	16.63290
H	15.64314	13.68400	17.14144
H	13.92797	14.22664	16.98508
H	15.26703	15.42276	16.95393
O	13.06708	8.43414	15.88499
H	12.84850	9.38963	15.66183
C	13.07974	8.25439	17.30334
H	13.10087	7.16570	17.51053
H	12.17575	8.68355	17.78763
H	13.99101	8.69661	17.76155

Intermediate 6

C	13.01077	9.75474	14.90161
C	13.19990	9.74323	16.28845
C	13.40320	8.54894	16.99464
C	13.44623	7.34755	16.28740
C	13.28827	7.33710	14.88612
C	13.05090	8.54428	14.20847
C	13.38339	6.01508	14.13626
O	13.06379	6.13680	12.74804

C	14.76330	5.39704	14.32944	C	10.10398	6.95782	15.50567
C	15.85735	6.14753	13.65989	H	10.51067	6.15973	16.15542
O	15.62098	7.11903	12.91782	H	10.65953	6.91141	14.54740
C	14.98523	4.31991	15.11262	H	9.03282	6.72425	15.29867
O	17.08415	5.71658	13.92600	O	10.50140	5.47609	12.49596
C	18.19061	6.51560	13.44228	H	11.42596	5.83707	12.39772
H	14.15126	3.84400	15.65426	C	9.54057	6.44363	12.08304
H	16.00109	3.92220	15.25598	H	8.53206	6.03568	12.29321
H	19.08438	5.87487	13.53236	H	9.60904	6.64142	10.99064
H	18.02951	6.81147	12.38879	H	9.63530	7.40939	12.62868
H	18.28152	7.40618	14.10064	O	10.92210	7.91448	18.68047
H	12.63263	5.32777	14.57041	H	10.79369	8.03804	17.68695
H	13.80768	6.63405	12.33032	C	10.44797	9.10773	19.29511
N	13.17293	11.00846	17.01588	H	10.44305	8.96741	20.39512
H	13.51563	8.54469	18.08202	H	9.40616	9.34313	18.97988
H	13.57667	6.40019	16.82918	H	11.08661	9.99169	19.06556
H	12.89013	8.54272	13.12479	O	10.74066	3.82394	14.54867
H	12.85490	10.70260	14.37972	H	10.52317	4.51931	13.85307
O	12.97584	12.05428	16.37769	C	11.43247	2.77402	13.87350
O	13.35392	10.98788	18.24418	H	11.69389	1.99294	14.61527
O	9.98230	9.95700	14.21699	H	12.37692	3.12067	13.39383
H	10.60962	10.03556	13.42761	H	10.79924	2.30531	13.08750
C	9.74321	11.27683	14.70072	O	11.46708	10.37195	12.07547
H	8.94343	11.23156	15.46761	H	12.41863	10.72146	12.14767
H	9.40017	11.95306	13.88688	C	11.40224	9.46450	10.98200
H	10.64506	11.72979	15.17476	H	10.33813	9.29184	10.72411
O	12.20866	4.34030	16.74231	H	11.86641	8.47546	11.21167
H	11.53650	4.17655	16.01968	H	11.90942	9.87560	10.08190
C	11.56657	4.45995	18.00160	O	13.27723	7.04766	19.67963
H	12.33724	4.67617	18.76963	H	12.38771	7.31101	19.29742
H	11.03407	3.52613	18.30180	C	13.59612	7.97776	20.70253
H	10.83119	5.29779	18.03873	H	14.64260	7.80265	21.03027
O	13.91594	11.27467	12.19183	H	12.94351	7.87250	21.60271
H	14.63031	10.65444	12.55921	H	13.53480	9.03871	20.36183
C	14.10185	12.55309	12.79780	O	15.78757	9.81148	13.29430
H	13.27340	13.21669	12.47769	H	15.70095	8.83059	13.17397
H	15.05715	13.02319	12.47394	C	17.13707	10.17405	13.00948
H	14.09344	12.50732	13.91090	H	17.24319	11.26372	13.17788
O	10.27120	8.20646	16.15389	H	17.41932	9.95503	11.95491
H	10.24806	8.92509	15.44295	H	17.84494	9.65072	13.68670

N	17.67905	8.36357	16.25561
C	16.87927	9.58343	16.47936
C	18.95869	8.48593	16.97799
C	16.93897	7.20392	16.78861
H	19.55154	7.56576	16.79330
H	19.52478	9.33653	16.54455
C	18.69159	8.69582	18.50514
H	15.94916	9.50312	15.88208
C	16.59791	9.75909	18.00778
H	17.44489	10.44752	16.07452
H	17.51579	6.28341	16.56564
C	16.69464	7.37770	18.32107
H	15.97763	7.13396	16.24432
H	19.17345	7.90146	19.11228
H	19.09548	9.66860	18.85482
H	16.98980	10.72855	18.37994
H	15.51190	9.74441	18.22941
N	17.23941	8.67317	18.77591
H	15.61301	7.33689	18.56761
H	17.19037	6.57183	18.90139

9) References

1 R. E. Plata and D. A. Singleton, J. Am. Chem. Soc., 2015, 137, 3811–3826.
2 J. Zhang and M. Dolg, Phys. Chem. Chem. Phys., 2015, 17, 24173–24181.
3 K. Vanommeslaeghe, E. Hatcher, C. Acharya, S. Kundu, S. Zhong, J. Shim, E. Darian, O. Guvench, P. Lopes, I. Vorobyov and A. D. Mackerell, J. Comput. Chem., 2010, 31, 671–690.
4 J. J. Stewart, J. Mol. Model., 2013, 19, 1–32.
5 J. J. Stewart, J. Comput. Aided Mol. Des., 1990, 4, 1–103.
6 J. P. Perdew, Phys. Rev. B, 1986, 33, 8822.
7 S. Grimme, S. Ehrlich and L. Goerigk, J. Comput. Chem., 2011, 32, 1456–1465.
8 F. Weigend and R. Ahlrichs, Phys. Chem. Chem. Phys., 2005, 7, 3297–3305.
9 F. Neese, Wiley Interdiscip. Rev. Comput. Mol. Sci., 2012, 2, 73–78.
10 A. D. Becke, J. Chem. Phys., 1993, 98, 5648–5652.
11 C. Riplinger and F. Neese, J. Chem. Phys., 2013, 138, 34106.
12 C. Riplinger, B. Sandhoefer, A. Hansen and F. Neese, J. Chem. Phys., 2013, 139, 134101.
13 P. Pinski, C. Riplinger, E. F. Valeev and F. Neese, J. Chem. Phys., 2015, 143, 34108.
14 C. Riplinger, P. Pinski, U. Becker, E. F. Valeev and F. Neese, J. Chem. Phys., 2016, 144, 24109.
15 J. P. Perdew, K. Burke and M. Ernzerhof, Phys. Rev. Lett., 1996, 77, 3865–3868.
16 A. V. Marenich, C. J. Cramer and D. G. Truhlar, J. Phys. Chem. B, 2009, 113, 6378–6396.
17 A. Klamt and G. Schüürmann, J. Chem. Soc. Perkin Trans. 2, 1993, 799–805.
18 C. J. Cramer, Essentials of Computational Chemistry: Theories and Models, Wiley, 2005.
19 TINKER 8: A Modular Software Package for Molecular Design and Simulation. Joshua A. Rackers, Marie L. Laury, Chao Lu, Zhi Wang, Louis Lagardère, Jean-Philip Piquemal, Pengyu Ren, Jay W. Ponder, 2017.
20 Grossfield, Alan. WHAM: An Implementation of the Weighted Histogram Analysis Method, version 2.0.9.1, http://membrane.urmc.rochester.edu/content/wham
